# Efficacy of Rituximab as Adjunctive Therapy to Immunosuppressive Agents in Adult Primary Focal Segmental Glomerulosclerosis

**DOI:** 10.1016/j.ekir.2025.08.046

**Published:** 2025-09-03

**Authors:** Osama Nady Mohamed, Marwa Ibrahim Mohamed, Shereen Mohammed Mohammed Elsaghir, Shimaa Abdelrazek, Aml Azzam, Tarek Mahmoud Senosy Mohamed, Hassan M.H. Mohammed, Rasha Yousef, Ayman Ahmed Abd Rabou, Reem Y. Abdelazeem Elgarhy, Alaa Khalifa Mohamed Mahdy, Mohamed Ahmed Abdelsamie, Manar M. Sayed, Eman Fathi, Nermeen Dahi Mohammed Toni, Rabeh Khairy Saleh, Basma Fathy

**Affiliations:** 1 Department of Internal Medicine, Faculty of Medicine, Minia University, Minia, Egypt; 2 Department of Cardiology, Faculty of Medicine, Minia University, Minia, Egypt; 3 Department of Clinical Pathology, Faculty of Medicine, Minia University, Minia, Egypt; 4 Department of Radiology, Faculty of Medicine, Minia University, Minia, Egypt; 5 Department of Public and Preventive Medicine, Faculty of Medicine, Minia University, Minia, Egypt; 6 Department of Pathology, Faculty of Medicine, Minia University, Minia, Egypt

**Keywords:** calcineurin inhibitors, focal segmental glomerulosclerosis, rituximab, steroid dependency, steroid resistance

## Abstract

**Introduction:**

Primary focal segmental glomerulosclerosis (FSGS) is a rare, likely immune-related disease. Rituximab (RTX) may help manage it, but existing adult data are limited and mainly based on case reports or case series. We aimed to evaluate RTX treatment outcomes in a large, multicenter adult cohort.

**Methods:**

This was a retrospective study of 160 patients with primary FSGS treated with RTX as adjunctive therapy to standard immunosuppressive agents between March 2015 and June 2023, that evaluated treatment responses at 3, 6, and 12 months, followed by an extended 24-month follow-up to assess long-term outcomes. Treatment outcomes included complete response (CR), partial response (PR), or no response. Treatment-related adverse effects and relapse-free survival (RFS) were evaluated at each visit. Patients undergoing RTX retreatment and those monitored for an extended 24-month period were analyzed independently.

**Results:**

Response rates at 3, 6, and 12 months were 57%, 71%, and 74%, respectively. Twelve-month RTX response was positively associated with lower baseline 24-hour proteinuria (odds ratio [OR] = 0.49, *P* = 0.043), steroid dependency (OR = 20.59, *P* = 0.002), and not otherwise specified (NOS) variant (OR = 234.19, *P* = 0.004), whereas it was negatively associated with calcineurin inhibitor (CNI) resistance (OR = 0.03, *P* = 0.01), treatment-related adverse effects (OR = 0.02, *P* = 0.003), and CD20+ B-cell repopulation at 6 months (OR = 0.08, *P* = 0.02). Thirty adverse effects occurred in 16 patients. Twelve-month responders had significantly longer RFS (mean: 32.8 months, 83.1% relapse-free) compared with nonresponders (median: 9 months, 16.7% relapse-free; *P* < 0.001). RTX retreatment was administered to 44 patients, with 2 achieving CR and 18 achieving PR. During long-term follow-up, 82 of 118 responders at 12 months maintained a sustained response, whereas 20 relapsed. Twenty-two of 42 nonresponders at 12 months had persistent disease despite RTX retreatment, whereas 10 achieved PR.

**Conclusion:**

RTX shows promise in treating primary FSGS, particularly in steroid-dependent patients, those with less severe nephrotic syndrome, and previous responders. RTX offers durable responses for most patients, but long-term management remains challenging in those who relapse and need retreatment.

## Graphical abstract

**Figure:**
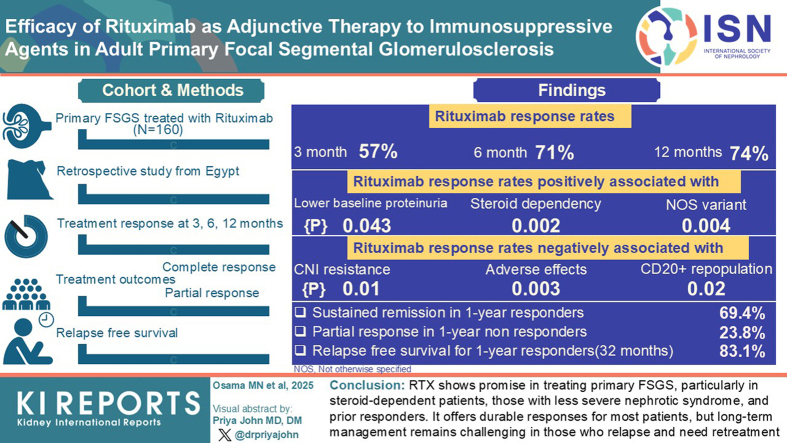

See Commentary on Page 3738

FSGS is a kidney disease that causes podocyte damage^1^ and is classified into 4 main types as follows: (i) primary FSGS, believed to be immune-related, involves a circulating permeability factor; (ii) secondary FSGS caused by systemic issues such as drugs, viruses, or maladaptive changes; (iii) genetic FSGS caused by sequence variation in podocyte or glomerular basement membrane proteins; and (iv) FSGS of unknown cause, with no identifiable trigger.^2^ Primary FSGS typically presents with nephrotic syndrome, diffuse podocyte foot process effacement on kidney biopsy electron microscopy, segmental scarring on light microscopy, and no immune deposits on immunofluorescence. Patients with severe nephrotic syndrome (proteinuria > 10 g/d) have a poor kidney prognosis and may develop end-stage kidney disease within 3 to 5 years, if unresponsive to treatment.^3^ Approximately one-third of these patients experience recurrent FSGS after kidney transplant, often leading to graft loss.^4^ The exact cause of primary FSGS is unclear; however, it may involve aberrant crosstalk between autoreactive T lymphocytes and B cells. A still undefined circulating permeability factor is believed to play a role.^5^ B cells appear to be involved as well, as evidenced in related podocytopathies.^6^ Despite immunosuppressive therapy, only ∼ 50% of patients achieve remission, and spontaneous remission is rare.^7^ Without effective treatment, 50% of patients progress to end-stage kidney disease within 6 to 8 years.^3^ Secondary FSGS is caused by identifiable factors, such as drugs, infections, or adaptive responses, and usually presents with subnephrotic proteinuria.^2^ Genetic FSGS, often resulting from podocyte or glomerular basement membrane mutations, can be familial or sporadic and is increasingly recognized in adults.^8^ Genetic testing is recommended primarily for adults with treatment resistance, a positive family history, or syndromic features.^9^

RTX is a monoclonal antibody that targets CD20 to treat immune disorders characterized by dysregulated B-cell activation.^10^^,^^11^ It may directly stabilize podocytes by interacting with SMPDL-3b and modulating acid sphingomyelinase, which is vital for podocyte structure and signaling.^12^ RTX shows promise for treating immune-related FSGS because of its potential to directly protect podocyte integrity, beyond its role in B-cell depletion. It has proven effective in pediatric patients with steroid-dependent (S-D) nephrotic syndrome.^13^ However, data on RTX in adult FSGS are limited and somewhat inconsistent.^14^ A few open-label trials and case reports have suggested that it is effective, with response rates ranging from 50% to 100% in S-D or calcineurin inhibitor (CNI)-sensitive cases.^15^, ^16^, ^17^ Previous studies on RTX in adults are limited by small sample sizes,^18^ single-center retrospective designs, and considerable variability in patient characteristics and definitions of remission. This study aimed to evaluate the effectiveness of RTX in inducing and maintaining remission in a larger cohort of adults with primary FSGS.

## Methods

### Study Design

This retrospective multicenter study included 160 patients selected from 3 tertiary university hospitals and 3 central hospitals located in Minia, Egypt. Hospital records spanning from March 2015 to June 2023 were reviewed to identify patients diagnosed with primary FSGS who had received RTX treatment and had ≥ 12 months of follow-up, followed by an extended 24-month period to assess long-term remission, relapse rates, and outcomes after retreatment when applicable. The study population comprised adult patients (aged > 18 years) with biopsy-proven primary FSGS, diagnosed through rigorous histopathological and clinical criteria specifically designed to differentiate primary from secondary FSGS and other causes of nephrotic syndrome. Histopathological diagnosis was established by the presence of segmental sclerosis affecting a subset of glomeruli on light microscopy, demonstrating the pathognomonic focal pattern (affecting < 50% of glomeruli) with segmental involvement (limited to portions of the glomerular tuft). Electron microscopy confirmation required extensive podocyte foot process effacement > 80%, whereas immunofluorescence typically showed negative staining or only nonspecific IgM and C3 deposition within sclerotic segments. Clinical presentation required fulfillment of nephrotic syndrome criteria, including proteinuria > 3.5 g/d and hypoalbuminemia (serum albumin < 3 g/dl).^2^ In addition, all enrolled patients had an estimated glomerular filtration rate (eGFR) ≥ 30 ml/min per 1.73 m^2^, calculated using the Chronic Kidney Disease-Epidemiology Collaboration equation, which provides more accurate GFR estimations.^19^ This GFR threshold was established based on the rationale that patients with substantially reduced GFR are more prone to having extensive glomerular and interstitial scarring, representing irreversible renal damage that would limit treatment responsiveness.

Patients were excluded from the study if their medical records indicated conditions associated with secondary FSGS related to hyperfiltration injury, including vesicoureteral reflux, morbid obesity, or reduced renal mass. The study also excluded individuals with a documented family history of kidney disease or those displaying syndromic characteristics. Further exclusion criteria included records indicating genetic variants of FSGS, ongoing infections, malignancy within the preceding 5 years, severe heart failure, cardiovascular manifestations, and type 1 or type 2 diabetes mellitus. Pregnant and lactating women were not included in the analysis, because RTX was not administered to these patients during standard clinical practice. This retrospective study involving human participants adhered to the Declaration of Helsinki and in accordance with the local ethical standards. All participants provided written informed consent, and the study protocol received approval from the hospital's Research Ethics Committee under Institutional Review Board approval number 1376-12-2024.

Of the 226 patients with FSGS retrospectively reviewed, 66 were systematically excluded through a 2-phase screening process. In phase 1, 36 patients were eliminated as follows: 26 with secondary FSGS because of morbid obesity (*n* = 8), renal agenesis (*n* = 6), uncontrolled hypertension (*n* = 8), and vesicoureteral reflux (*n* = 4), as well as 10 patients with a family history of renal disease. The remaining 190 patients with primary FSGS had previously received steroid therapy, with outcomes documented as 110 steroid-resistant (SR) and 80 S-D cases. In phase 2, records showed that 110 SR patients had undergone targeted next-generation sequencing during their clinical evaluation, leading to the exclusion of 20 with identified genetic mutations. In addition, 10 S-D patients were excluded because of documented contraindications to RTX therapy, including heart failure (*n* = 6) and active infections (*n* = 4). The final cohort consisted of 160 patients with primary FSGS who had been treated with RTX therapy during the course of their clinical care (90 SR and 70 S-D).

### Comprehensive Clinical, Laboratory, and Imaging Workup

All clinical and laboratory data were retrospectively obtained from patients’ medical records, including previously documented history, physical examination findings, and investigation results recorded at the time of diagnosis and follow-up. A comprehensive clinical history and complete systemic physical examination were obtained and analyzed for all participants. The extracted medical history encompassed demographic factors, including age and sex, as well as relevant clinical variables such as smoking, diabetes mellitus, hypertension, and specific cardiovascular manifestations. The physical examination findings, as documented in patient charts, involved a systematic assessment of vital signs and body mass index (BMI), followed by a detailed evaluation of the abdominal, cardiovascular, neurological, and pulmonary systems. This comprehensive examination was conducted to identify and exclude secondary causes of FSGS, including congenital abnormalities (low birthweight, renal dysplasia, or agenesis), acquired conditions (reflux nephropathy and surgical renal ablation), metabolic disorders (obesity, poorly controlled diabetes mellitus, and uncontrolled hypertension), and medication-induced FSGS (such as that caused by anabolic steroids, interferon, lithium, or sirolimus). Laboratory results documented in the medical records were reviewed to confirm the diagnosis of primary FSGS and to assess clinical suitability for RTX therapy at the time of its administration (Supplementary Methods).

Echocardiography data were retrieved from reports of comprehensive 2- and 3-dimensional transthoracic echocardiograms performed before RTX therapy during routine clinical evaluation. These records were reviewed to identify heart failure, congenital cyanotic heart disease (as potential secondary causes of FSGS), and any documented cardiac abnormalities, including myocarditis, that could be relevant to RTX therapy.^19^^,^^20^ Kidney biopsy findings were obtained from pathology reports of percutaneous biopsies performed using automated biopsy guns under ultrasound guidance. Tissue samples were processed for light microscopy and electron microscopy according to standard hospital protocols^21^^,^^22^ (Supplementary Figure S1). Abdominal ultrasound findings were obtained from radiology reports, including documented assessments for renal dysplasia, agenesis, and previous surgical renal ablation. Renal Doppler ultrasound findings were also retrieved from these reports, which included evaluations for renal artery stenosis. According to the records, renal Doppler ultrasound had been performed using a 5 MHz convex transducer on a LOGIQ E9 ultrasound machine (GE Healthcare, Chalfont St Giles, UK) with patients in the supine position. Color Doppler criteria for stenosis, as documented, included a peak systolic velocity ≥ 180 cm/s, a renal-to-interlobar ratio > 5, and a resistive index difference between kidneys > 5% (Supplementary Methods).

### Immunosuppression Therapy Before and After RTX Administration

All patients had initially received glucocorticoid therapy and were categorized as SR (*n* = 90) or S-D (*n* = 70) based on documented treatment outcomes. SR FSGS was defined as failure to achieve complete remission after 16 weeks of prednisone or prednisolone at 60 mg/m^2^/d (or 2 mg/kg every other day), whereas S-D FSGS was defined as relapse during tapering or within 14 days of discontinuation. Most patients were subsequently prescribed CNIs (*n* = 116) as second-line therapy, whereas mycophenolate mofetil (MMF) (*n* = 44) was used in patients with eGFR < 40 ml/min per 1.73 m^2^. CNI resistance was defined as persistent nephrotic-range proteinuria after ≥ 16 weeks of therapy at therapeutic levels, whereas dependence was defined as relapse during or shortly after stopping CNIs following ≥ 12 months of use. Glucocorticoids were continued during second-line therapy and tapered according to documented protocols once remission was achieved. In S-D FSGS, tapering was recorded as beginning after 4 to 8 weeks of stable remission, starting from a maintenance dose of 0.5 to 1 mg/kg/d, with reductions of 2.5 to 5 mg every 1 to 2 weeks to reach 5 to 10 mg/d within 2 to 3 months, then further reduced by 1 to 2.5 mg every 2 to 4 weeks until discontinuation over 6 to 12 months. In SR FSGS, tapering was typically faster, with documented schedules involving reductions of 5 to 10 mg/wk to 5 to 10 mg/d, or complete withdrawal within 2 to 3 months.^2^

RTX had been administered in patients with MMF or CNI resistance, CNI dependence, or frequent relapses. In responders, tacrolimus was generally maintained at 0.05 to 0.1 mg/kg/d (target trough: 3–7 ng/ml), and cyclosporine A at 2 to 3 mg/kg/d (target: 75–150 ng/ml). CNIs were tapered by reducing doses 10% to 20% every 4 to 8 weeks to reach trough levels < 5 ng/ml for tacrolimus and < 75 ng/ml for cyclosporine. MMF was maintained at 500 to 1000 mg twice daily (1–2 g/d), with tapering initiated after 6 months in remission by reducing 250 to 500 mg every 4 to 6 weeks to 750 mg, then 500 mg, before discontinuation. If remission persisted without relapse or B-cell repopulation, complete withdrawal of CNIs and MMF was considered after 12 to 18 months. In nonresponders to RTX, immunosuppression was maintained or intensified according to clinical documentation. Tacrolimus was increased to 0.1 to 0.15 mg/kg/d (target trough: 5–10 ng/ml), cyclosporine to 3 to 5 mg/kg/d (target: 100–200 ng/ml), and MMF was continued at full dose (1000–1500 mg twice daily, 2–3 g/d). In these cases, tapering was not recommended due to ongoing disease activity.^2^

### RTX Treatment Protocols

All RTX treatment schedules documented as having been administered within 4 weeks, including 375 mg/m^2^/wk and 2 doses of 1 g given 2 weeks apart, were regarded as RTX induction therapy. Medical records indicated that all patients had received preinfusion treatment with diphenhydramine 50 mg orally (30–60 minutes before RTX), acetaminophen 1000 mg orally (30–60 minutes before), and methylprednisolone 100 mg diluted in 0.9% sodium chloride to a total volume of 50 ml, infused at 200 ml/h and completed 30 minutes before the start of RTX infusion. Patients were categorized in the records at the time of the initial RTX dose according to the documented indication for treatment: persistent disease activity or relapse. A relapse was defined in the records as a return of proteinuria > 3.5 g/d following either a PR or CR. Persistent disease activity was recorded as proteinuria > 3.5 g/d without ever achieving PR (i.e., a reduction < 3.5 g/24 h).^18^

Peripheral blood CD20^+^ B-cell counts were obtained from flow cytometry reports performed at approximately 3, 6, and 12 months after RTX administration, with repopulation defined in laboratory records as CD20^+^ B-cell counts > 0 × 10^6^ cells/l.^11^ Patients documented as having undergone RTX retreatment were analyzed separately. Those with 24-month follow-up data were included in the long-term analysis, regardless of additional RTX infusions.

### Response to RTX Treatment (Study Outcomes)

The study primarily aimed to assess the effectiveness of RTX-based protocols for achieving remission in patients with primary FSGS. Serum creatinine, 24-hour proteinuria, serum albumin, and eGFR values, documented on day 0 (immediately before RTX administration) and at approximately 3, 6, and 12 months posttreatment, were retrieved from patient records. Treatment response was evaluated at 3, 6, and 12 months following RTX, as well as throughout the 24-month follow-up period. Response was subdivided into CR and PR, in accordance with the standard definitions used in nephrotic syndrome studies. CR was defined as a proteinuria level < 0.3 g/d (or a urine protein-to-creatinine ratio < 300 mg/g), with stable serum creatinine and serum albumin exceeding 3.5 g/dl. PR was defined as a proteinuria level between 0.3 and 3.5 g/d (or a urine protein-to-creatinine ratio between 300 and 3500 mg/g), showing a reduction ≥ 50% from baseline, along with stable kidney function (i.e., no worsening of serum creatinine).^2^ Patients who did not meet the aforementioned criteria were classified as nonresponders. According to the clinical documentation, a second course of RTX in primary FSGS was considered in patients who relapsed after initial remission,^23^ exhibited persistent nephrotic-range proteinuria,^24^ or showed peripheral B-cell reconstitution (e.g., CD20+ recovery),^25^ because these factors were strongly associated with disease recurrence. RFS was defined in the clinical records as the interval from the date of initial RTX administration to a documented clinical relapse or the last recorded follow-up in patients who remained in remission.^26^ Information on symptoms and potential treatment-related adverse effects was obtained from clinical notes recorded during follow-up visits after RTX administration. Any incident necessitating hospitalization, i.v. treatments (e.g., antibiotics or diuretics), a cancer diagnosis, infection, infusion-related reaction, cardiac complications (such as myocarditis), or death was recorded as a serious adverse event. Clinic visits documented detailed medical history, physical examination findings, and results of investigations performed as part of routine evaluation, including complete blood count, liver and renal function tests, fasting blood glucose, C-reactive protein, serum albumin, urinalysis, 24-hour urinary protein quantification, chest X-ray, and echocardiography.

### Statistical Analysis

Data analysis was conducted using IBM SPSS Statistics for Windows, Version 25.0 (IBM Corp., Armonk, NY). Categorical variables were presented as frequencies and percentages, whereas continuous variables were expressed as median with interquartile range or mean with SD. Chi-square testing was applied for the analysis of categorical variables. For 2-group comparisons, the Mann–Whitney U test and independent *t* test were used for nonparametric and parametric continuous variables, respectively. The paired *t* test was used to assess temporal changes in serum creatinine, serum albumin, and 24-hour proteinuria. Both univariate and multivariate logistic regression analyses were performed to identify clinical, histopathological, and laboratory factors predictive of treatment response at 12 months post-RTX administration. Survival analysis was performed using the Kaplan–Meier method to estimate RFS, and differences between groups were compared using the log-rank test.

## Results

### Baseline Characteristics of the Study Population

This study analyzed 160 patients with primary FSGS treated with RTX as adjunctive therapy to standard immunosuppressive agents, comprising predominantly young adult males (mean age: 28.29 years, 63% male) with severe disease burden, including significant proteinuria (8.32 g/d), hypoalbuminemia (2.17 g/dl), and moderately impaired kidney function (eGFR: 81.2 ml/min per 1.73 m^2^). The cohort represented treatment-resistant disease with 56% SR and 44% S-D patients, whereas 72% had previous CNI exposure (40% CNI-dependent, 25% frequent relapsers, 7% CNI-resistant) and 28% had MMF resistance. Histologically, NOS variant predominated (61%), followed by cellular (26%), collapsing (9%), and tip variants (4%). RTX was administered as 1 g 2 weeks apart (47%) or 375 mg weekly (53%) protocols, with 10% experiencing adverse effects, including infections (5%), hypersensitivity reactions (4%), and cardiotoxicity (1%) (Table 1).

**Table 1:** Baseline characteristics of the study population

Variables	Characteristics of the study population (*n* = 160)
Age (yr)	28.29 ± 7.15 (18.00–41.00)
Sex, *n* (%)	
Male	100 (63%)
Female	60 (37%)
SBP (mm Hg)	138.81 ± 19.13 (110.00–170.00)
DBP (mm Hg)	84.06 ± 14.77 (65.00–100.00)
HTN, *n* (%)	72 (45%)
Smoking, *n* (%)	48 (30%)
BMI (kg/m^2^)	25.00 ± 2.33 (20.76–30.46)
Time interval between FSGS diagnosis and RTX initiation (mo)	34.65 ± 17.24 (12.00–80.00)
Relapses before RTX initiation, *n*	212
Serum creatinine (mg/dl)	1.50 ± 0.87 (0.60–3.00)
eGFR (ml/min per 1.73 m^2^)	81.20 ± 44.52 (30.00–143.00)
Serum albumin (g/dl)	2.17 ± 0.59 (1.30–3.10)
24-h proteinuria (g/d)	8.32 ± 3.67 (3.50–15.00)
TC (mg/dl)	332.80 ± 61.28 (235.00–436.00)
TG (mg/dl)	211.63 ±37.66 (175.00–310.00)
RTX dosing, *n* (%)	
1 g 2 wks apart	76 (47%)
375 mg every week	84 (53%)
Steroid status, *n* (%)	
SR	90 (56%)
S-D	70 (44%)
Steroid state before RTX initiation	
Steroid dose	5.69 ± 11.15 (0.0–30.00)
Steroid discontinuation, *n* (%)	126 (79%)
Concomitant steroid therapy, *n* (%)	34 (21%)
Prior and concomitant immunosuppressive therapies, *n* (%)	
CNIs	116 (72%)
MMF	44 (28%)
Response to CNIs	
CNIs resistant	12 (7%)
CNIs dependent	64 (40%)
Frequent relapse on CNIs	40 (25%)
Response to MMF	
MMF resistant	44 (28%)
Histological variants, *n* (%)	
NOS	98 (61%)
Cellular	42 (26 %)
Collapsing	14 (9 %)
Tip	6 (4%)
Immunosuppressive therapies after RTX	
Steroid dose after RTX initiation	4.6 ± 4.84
Steroid discontinuation after RTX initiation, *n* (%)	12 (37%)
Immunosuppressive therapy discontinuation, *n* (%)	77 (54%)
RTX indication, *n* (%)	
Persistent disease activity	80 (50%)
Relapses	80 (50%)
Patients experiencing RTX adverse side effects, *n* (%)	16 (10%)
Infection	8 (5%)
Hypersensitivity reaction	6 (4%)
Cardiotoxicity	2 (1%)

BMI, body mass index; CNIs, calcineurin inhibitors; DBP, diastolic blood pressure; eGFR, estimated glomerular filtration rate; FSGS, focal segmental glomerulosclerosis; HTN, hypertension; MMF, mycophenolate mofetil; NOS, not otherwise specified; RTX, rituximab; SBP, systolic blood pressure; S-D, steroid-dependent; SR, steroid-resistant; TC, total cholesterol; TG, total triglycerides.

Continuous variables are expressed as mean ± SD (minimum–maximum). Categorical variables are expressed as number (percentage).

### Response to RTX Treatment

Response rates at 3, 6, and 12 months were 57%, 71%, and 74%, respectively. At 3, 6, and 12 months, we reported CR in 40%, 45%, and 34% of patients, respectively; whereas PR was found in 17%, 26%, and 40% of patients (Table 2). Based on 12-month response following the first RTX administration, patients were classified into responders and nonresponders with similar median ages (28 vs. 27 years) and comparable sex distribution (61% vs. 67% male). Nonresponders demonstrated significantly worse baseline disease parameters, including higher systolic and diastolic blood pressures (*P* = 0.001 and *P* = 0.003), serum creatinine (*P* < 0.001), and 24-hour proteinuria levels (*P* < 0.001), alongside lower eGFR and serum albumin (both *P* = 0.001). Histological variants differed markedly, with NOS variant predominating in responders (75% vs 24%, *P* < 0.001), whereas nonresponders had higher frequencies of cellular (43% vs. 20%, *P* = 0.004) and collapsing (33% vs. 0%, *P* < 0.001) variants. RTX adverse effects were significantly more common in nonresponders (29% vs. 3%, *P* < 0.001), whereas age, sex, BMI, time to RTX initiation, and dosing regimens showed no significant differences between groups (Table 3).

**Table 2:** Response rates at months 3, 6, and 12 after RTX administration

Variables	3 mos	6 mos	12 mos
NR, *n* (%)	68 (43%)	46 (29%)	42 (26%)
CR, *n* (%)	64 (40%)	72 (45%)	54 (34%)
PR, *n* (%)	28 (17%)	42 (26%)	64 (40%)

CR, complete response; NR, no response; PR; partial response; RTX, rituximab.

Data are expressed as *n* (%).

**Table 3:** Baseline and posttreatment characteristics according to 12-month RTX response

Variables	Responders *n* = 118	Nonresponders *n* = 42	*P*
Age (yr)	28.00 (35.00–22.00)	27.00 (33.75–23.5)	0.94
Sex, *n* (%)			
Male	72 (61%)	28 (67%)	0.52
Female	46 (39%)	14 (33%)	
SBP (mm Hg)	130.00 (120.00–160.00)	155.00 (142.5–158.75)	0.001
DBP (mm Hg)	80.00 (70.00–100.00)	100.00 (87.5–100.00)	0.003
Smoking, *n* (%)	34 (29%)	14 (33%)	0.58
BMI (kg/m^2^)	25. 04 ± 2.39	24.89 ± 2.16	0.71
HTN, *n* (%)	40 (34%)	32 (76%)	< 0.001
Time interval between FSGS diagnosis and RTX initiation (mo)	35.20 ± 17.53	33.09 ± 16.47	0.49
Baseline serum creatinine (mg/dl)	0.90 (1.9–0.8)	2.10 (2.85–1.43)	< 0.001
Baseline eGFR (ml/min per 1.73 m^2^)	107.00 (124.00–46.00)	36.00 (68.75–26.5)	0.001
Baseline serum albumin (g/dl)	2.40 (2.9–1.7)	1.90 (2.00–1.65)	0.001
Baseline 24-h proteinuria (g/d)	6.70 (10.00–4.00)	10.90 (12.75–9.00)	< 0.001
TC (mg/dl)	345.00 (366.00–290.00)	345.00 (364.00–224.00)	0.6
TG (mg/dl)	200.00 (235.00–185.00)	178.00 (242.5–175.00)	0.003
RTX dosing, *n* (%)			
1 g 2 wks apart	54 (46%)	22 (52%)	0.46
375 mg every week	64 (54%)	20 (48%)	0.46
Steroid status, *n* (%)			
SR	58 (49%)	32 (76%)	0.002
S-D	60 (51%)	10 (24%)	
Steroid dose before RTX initiation	4.24 ± 10.12	9.76 ± 12.92	0.004
Steroid discontinuation before RTX initiation, *n* (%)	100 (85%)	26 (62%)	0.002
Prior and concomitant immunosuppressive therapies, *n* (%)			
CNIs	96 (81%)	20 (48%)	< 0.001
MMF	22 (19%)	22 (52%)	
Response to CNIs, *n* (%)			
CNIs resistant	4 (3%)	8 (19%)	0.001
CNIs dependent	58 (49%)	6 (14%)	< 0.001
Frequent relapse on CNIs	34 (29%)	6 (14%)	0.06
Response to MMF, *n* (%)			
MMF resistant	22 (19%)	22 (52%)	< 0.001
Response to CNI and MMF combination	54 (46%)	14 (33%)	0.16
Histological variants, *n* (%)			
NOS	88 (75%)	10 (24%)	< 0.001
Cellular	24 (20%)	18 (43%)	0.004
Collapsing	0.0 (0%)	14 (33%)	< 0.001
Tip	6 (5%)	0.0 (0%)	0.14
RTX indication, *n* (%)			
Persistent activity	66 (56%)	14 (33%)	0.01
Relapses	52 (44%)	28 (67%)	
Immunosuppressive therapy after RTX			
Steroid dose after RTX initiation	1.88 ± 2.5	7.34 ± 5.12	0.001
Steroid discontinuation after RTX initiation, *n* (%)	10 (63%)	2 (13%)	0.003
Discontinuation of immunosuppressive therapy	77 (76%)	0.0 (0%)	< 0.001
Patients experiencing RTX adverse side effects, *n* (%)	4 (3%)	12 (29%)	< 0.001

BMI, body mass index; CNIs, calcineurin inhibitors; DBP, diastolic blood pressure; eGFR, estimated glomerular filtration rate; HTN, hypertension; MMF, mycophenolate mofetil; NOS, not otherwise specified; RTX, rituximab; SBP, systolic blood pressure; S-D, steroid-dependent; SR, steroid-resistant; TC, total cholesterol; TG, total triglycerides.

Continuous variables are expressed as median (interquartile range) or mean (SD). Categorical variables are expressed as number (percentage).

Subgroup analysis demonstrated that patients with less severe baseline disease parameters achieved superior 12-month RTX response rates, including those with proteinuria < 5 g/24 h (100% vs. 63%), serum albumin ≥ 2.5 g/dl (100% vs. 61%), and eGFR ≥ 60 ml/min per 1.73 m^2^ (89% vs. 52%). S-D patients responded more favorably than SR patients (86% vs. 64%), and CNI-dependent patients significantly outperformed CNI-resistant patients (91% vs. 33%). Histological variants substantially influenced treatment outcomes, with tip and NOS variants achieving the highest response rates (100% and 90%, respectively) compared with cellular (57%) and collapsing (0%) variants. RTX dosing demonstrated minimal impact on treatment efficacy, with comparable response rates between high-dose and low-dose groups (71% vs. 76%). Patients with pediatric-onset FSGS demonstrated superior early RTX response rates at 3 months (80% vs. 52%, *P* = 0.006) and 6 months (100% vs. 65%, *P* < 0.001) compared with patients with adult-onset FSGS; however, this advantage disappeared by 12 months, where adult patients achieved numerically higher response rates (75% vs. 67%, *P* = 0.33), suggesting faster onset but comparable ultimate efficacy between age groups (Table 4). BMI and cumulative RTX dose had no significant impact on treatment response at 3-, 6-, and 12-month intervals. However, steroid discontinuation at RTX initiation was significantly associated with improved response, with 85% of 12-month responders having discontinued steroids compared with only 15% who continued steroid therapy (*P* = 0.002) (Supplementary Table S1).

**Table 4:** Response rates at 3, 6, and 12 months in different study subgroups in relation to different baseline characteristics

Subgroups	Response		
	3 mos	6 mos	12 mos
Proteinuria ≥ 5 g/24 h (*n* = 112)	44 (39%)	66 (59%)	70 (63%)
Proteinuria < 5 g/24 h (*n* = 48)	48 (100%)	48 (100%)	48 (100%)
Serum albumin ≥ 2.5 g/dl (*n* = 52)	52 (100%)	52 (100%)	52 (100%)
Serum albumin < 2.5 g/dl (*n* = 108)	40 (37%)	62 (57%)	66 (61%)
eGFR ≥ 60 ml/min per 1.73 m^2^ (*n* = 94)	70 (75%)	80 (85%)	84 (89%)
eGFR < 60 ml/min per 1.73 m^2^ (*n* = 66)	22 (33%)	34 (51%)	34 (52%)
Cumulative RTX dose ≥ 2 g (*n* = 76)	44 (58%)	52 (68%)	54 (71%)
Cumulative RTX dose < 2 g (*n* = 84)	48 (57%)	62 (74%)	64 (76%)
Childhood-onset FSGS (*n* = 30)	24 (80%)	30 (100%)	20 (67%)
Adult-onset FSGS (*n* = 130)	68 (52%)	84 (65%)	98 (75%)
Steroid dependency (*n* = 70)	46 (66%)	64 (91%)	60 (86%)
Steroid resistance (*n* = 90)	46 (51%)	50 (56%)	58 (64%)
CNI resistance (*n* = 12)	4 (33%)	6 (50%)	4 (33%)
CNI dependence (*n* = 64)	50 (78%)	56 (88%)	58 (91%)
Frequent relapse on CNI (*n* = 40)	22 (55%)	30 (75%)	34 (85%)
Histological variants			
NOS	78/98 (80%)	88/98 (90%)	88/98 (90%)
Cellular	8/42 (19%)	20/42 (48%)	24/42 (57%)
Collapsing	0/14 (0%)	0/14 (0%)	0/14 (0%)
Tip	6/6 (100%)	6/6 (100%)	6/6 (100%)

CNI, calcineurin inhibitor; eGFR, estimated glomerular filtration rate; FSGS, focal segmental glomerulosclerosis; NOS, not otherwise specified; RTX, rituximab.

Data are expressed as *n* (%).

**Figure 1:**
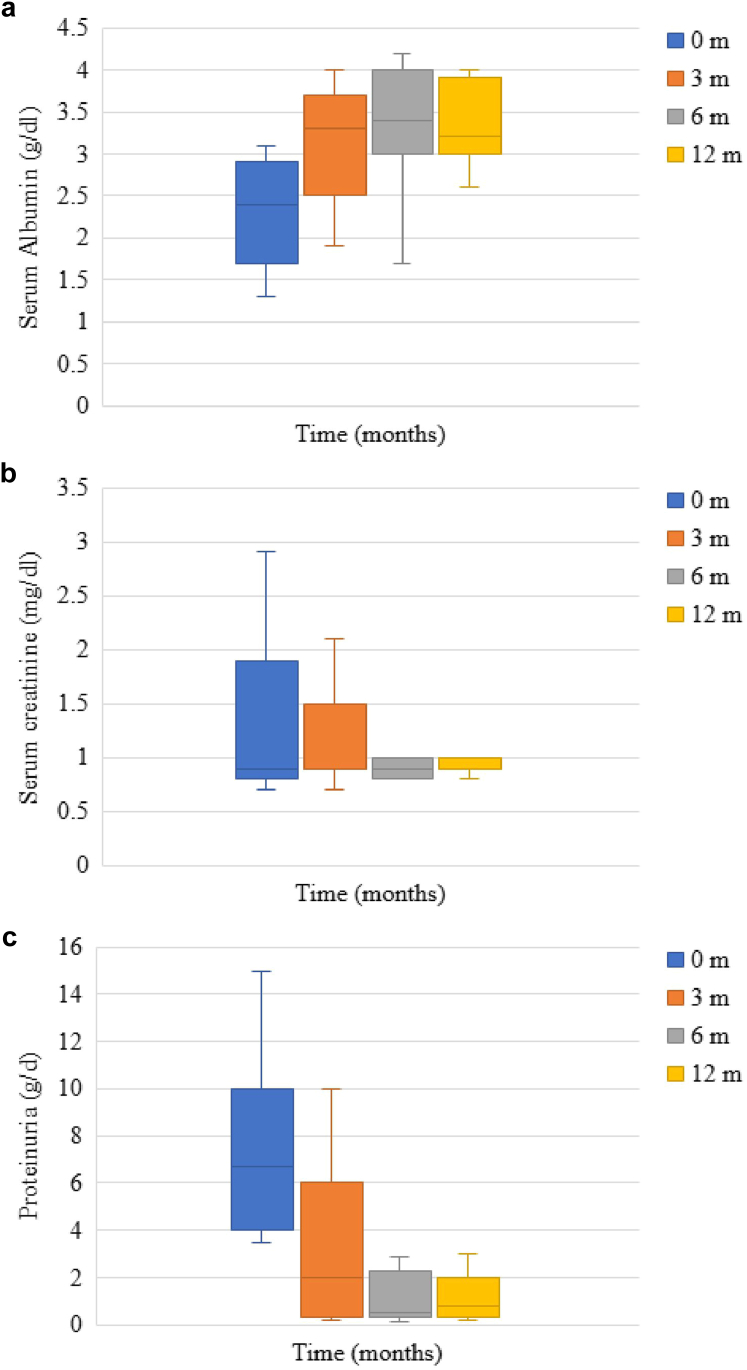
Changes in proteinuria, serum albumin, and serum creatinine in responders to RTX at 12 months. (a) Serum albumin levels increased considerably compared with baseline level at 3, 6, and 12 months (*P <* 0.001 for each), from a baseline median value of 2.4 g/dl (range: 1.3–3.1) to a maximum median value of 3.4 g/dl (range: 1.7–4.2) at 6 months. (b) Serum creatinine level was found to be substantially decreased at 12 months compared with baseline creatinine (*P =* 0.002). (c) Proteinuria was significantly decreased at all time points in comparison with baseline level (*P* < 0.001 for each), with the lowest value reached at 6 months (median 0.5 g/24 h, range: 0.1–6). m, months; RTX, rituximab.

**Figure 2:**
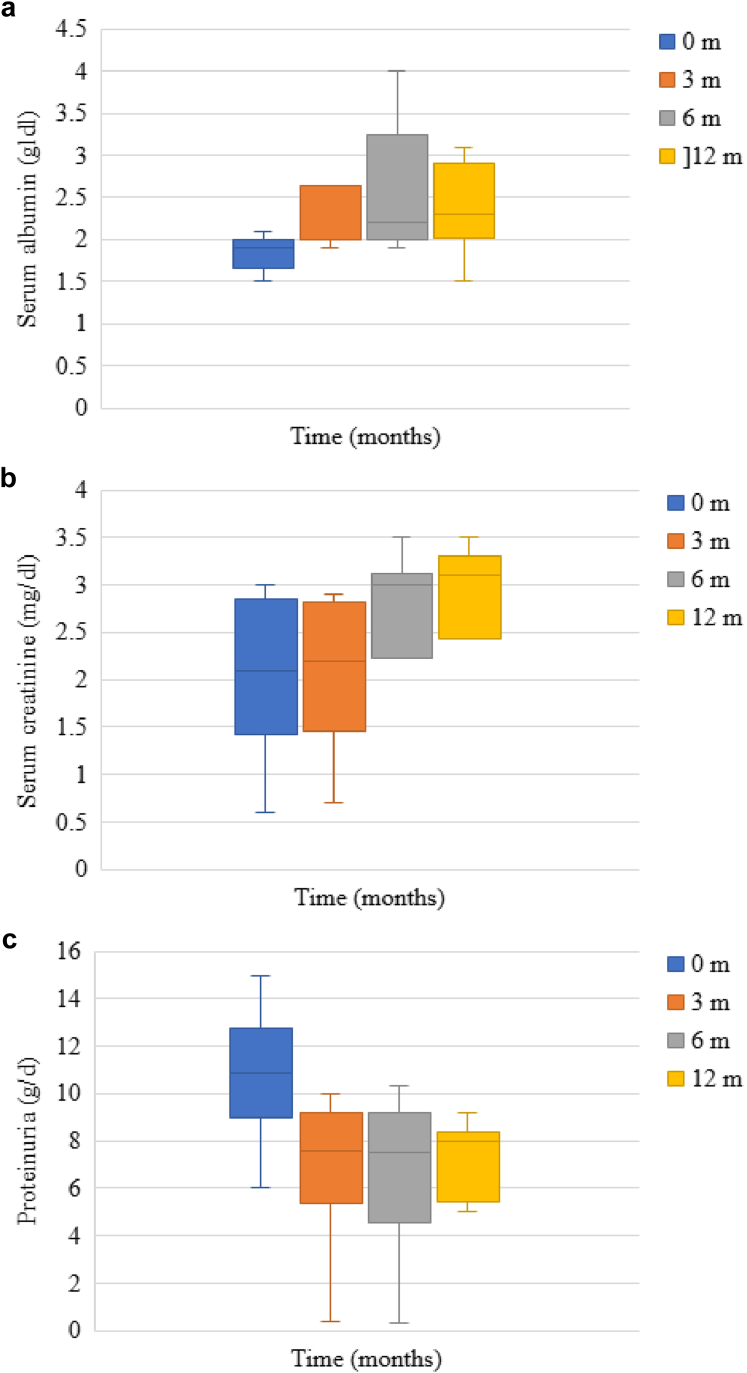
Changes in proteinuria, serum albumin, and serum creatinine in nonresponders to RTX at 12 months. (a) Serum albumin levels increased considerably compared with baseline level at 3, 6, and 12 months (*P* < 0.001, for each), with a maximum median value of 2.3 g/dl at 12 months (range 1.5–3.1). (b) Serum creatinine level was found to be substantially increased at 6 and 12 months compared with baseline creatinine (*P* < 0.001 for each). (c) Proteinuria was significantly decreased at all time points in comparison to baseline level (*P* < 0.001 for each), with the lowest value reached at 6 months (median: 7.5 g/24 h, range: 0.3–10.3). m, months; RTX, rituximab.

RTX treatment resulted in significant biochemical improvements across all time points, with serum albumin levels showing substantial increases at 3, 6, and 12 months compared with baseline (*P* < 0.001 for each time point), whereas serum creatinine remained stable throughout the follow-up period. Proteinuria demonstrated significant reduction at all evaluation points versus baseline (*P* < 0.001 for each), achieving its nadir at 12 months (mean: 2.73 ± 2.88 g/d, range: 0.2–9.2) (Supplementary Figure S2). Whereas RTX responders at 12 months experienced marked proteinuria improvement with normalization of albumin levels during follow-up (Figure 1), even patients classified as nonresponders at 12 months showed measurable biochemical improvement (Figure 2).

### Immunosuppressive Therapy Before and After RTX Initiation

The analysis of previous immunosuppressive therapy revealed significant differences between responders and nonresponders, suggesting that treatment resistance patterns influenced RTX efficacy. Responders had a substantially higher rate of previous CNI exposure (81% vs. 48%, *P* < 0.001), whereas nonresponders showed greater previous MMF usage (52% vs. 19%, *P* < 0.001). More importantly, the response patterns to previous therapies were distinctly different between groups: nonresponders demonstrated higher rates of CNI resistance (19% vs. 3%, *P* = 0.001), indicating a more refractory disease phenotype. Conversely, responders showed higher rates of CNI dependence (49% vs. 14%, *P* < 0.001), suggesting their disease was more amenable to immunosuppressive control. The steroid response patterns further supported this trend, with nonresponders having significantly higher rates of steroid resistance (76% vs. 49%, *P* = 0.002) and higher baseline steroid doses (*P* = 0.004). Notably, a significantly higher proportion of responders had discontinued steroids before RTX initiation (85% vs. 62%, *P* = 0.002), suggesting that successful steroid weaning may be a positive prognostic indicator for subsequent RTX response. Combination CNI and MMF therapy was administered to 46% of responders versus 33% of nonresponders (*P* = 0.16). RTX therapy significantly facilitated steroid tapering and withdrawal, with responders showing a lower mean steroid dose (1.88 ± 2.5 mg/d) compared with nonresponders (7.34 ± 5.12 mg/d, *P* = 0.001), and 63% of responders successfully discontinuing steroids versus 13% of nonresponders (*P* = 0.003). These findings underscore the steroid-sparing effect of RTX and its role in improving long-term disease control and patient safety. A significantly higher proportion of RTX responders discontinued immunosuppressive therapy following response compared with nonresponders (76% vs. 0%, *P* < 0.001), suggesting that achieving remission with RTX facilitated successful withdrawal of concurrent immunosuppressive agents (Table 3).

### Adverse Side Effects of RTX in the Study Population

A total of 30 adverse events were recorded in 16 patients, with infections being the most common (16 events, 54%), followed by infusion-related reactions (10 events, 33%) and myocarditis (4 events, 13%). Adverse events were significantly more prevalent among nonresponders (12 patients) compared with responders (4 patients) (*P* < 0.001). During the 24-month follow-up period, 10 of the 16 patients who experienced adverse events developed persistent nephrotic-range proteinuria with subsequent relapse, whereas the remaining 6 achieved PR. No deaths were reported during the follow-up period (Supplementary Table S2).

### CD20+ B-cell recovery patterns and their association with RTX treatment outcomes

This study revealed a strong inverse relationship between CD20+ B-cell repopulation timing and RTX treatment success. Patients with early B-cell repopulation (at 3 months) showed complete treatment failure with 0% response rates throughout 12 months, whereas those without repopulation achieved 100% response rates. The pattern continued with 6-month repopulation showing poor responses (6% at 3 months, 5% at 6 months, and 5% at 12 months) versus excellent responses (94%, 95%, and 95%, respectively) in nonrepopulated patients, and even 12-month repopulation significantly reduced treatment efficacy (13% at 3 months, 10% at 6 months, and 5% at 12 months versus 87%, 90%, and 95%, respectively, in nonrepopulated patients). All differences were statistically significant (*P* < 0.001), demonstrating that earlier B-cell repopulation predicted worse RTX treatment outcomes, with the timing of repopulation serving as a crucial predictor of therapeutic success (Supplementary Table S3).

### Factors predicting RTX response at 12 months

Univariate logistic regression identified several significant predictors of 12-month RTX response (Supplementary Table S4), with significantly associated factors subsequently analyzed in multivariate modeling to determine independent predictors of treatment outcomes. Multivariate analysis demonstrated that lower baseline 24-hour proteinuria (OR = 0.49, *P* = 0.043), steroid dependency (OR = 20.59, *P* = 0.002), and the NOS histological variant (OR = 234.19, *P* = 0.004) were independent predictors of higher odds of 12-month RTX response. Conversely, factors independently associated with lower odds of response included CNI resistance (OR = 0.03, *P* = 0.01), RTX indication for frequent relapses (OR = 0.18, *P* = 0.02), treatment-related adverse effects (OR = 0.02, *P* = 0.003), and CD20^+^ B-cell repopulation at 6 months (OR = 0.08, *P* = 0.02) (Table 5).

**Table 5:** Multivariate logistic regression analysis identifying predictors of 12-month RTX response

Variables	Multivariate logistic regression			
	*P*	Odds ratio	95% CI	
			Lower bound	Uppper bound
Baseline eGFR	0.16	0.98	0.95	1.01
Baseline serum albumin	0.098	8.27	0.68	101.04
Baseline proteinuria	0.043	0.49	0.01	0.21
Steroid status (steroid dependency)	0.002	20.59	2.89	146.36
CNI status (CNI dependence)	0.97	1.04	0.15	7.35
CNI resistance	0.01	0.03	0.002	0.48
MMF resistance	0.22	0.24	0.02	2.36
RTX indication (frequent relapses)	0.02	0.18	0.04	0.79
Histological variant (NOS)	0.004	234.19	5.81	9435.59
Histological variant (cellular)	0.052	13.13	1.01	170.07
RTX side effects	0.003	0.02	0.001	0.24
CD20+ B-cell repopulation at 6 mos	0.02	0.08	0.01	0.66

CI, confidence interval; CNI, calcineurin inhibitor; eGFR, estimated glomerular filtration rate; MMF, mycophenolate mofetil; NOS, not otherwise specified; OR, odds ratio; RTX, rituximab.

Per 1 ml/min per 1.73 m^2^ increase in eGFR, there was no significant change in the odds of response. Furthermore, per 1 g/dl increase in baseline serum albumin, there was no significant positive association with the odds of response. Conversely, for each 1 g/d increase in proteinuria, the odds of response significantly decreased.

### Factors influencing RFS After RTX treatment

**Figure 3:**
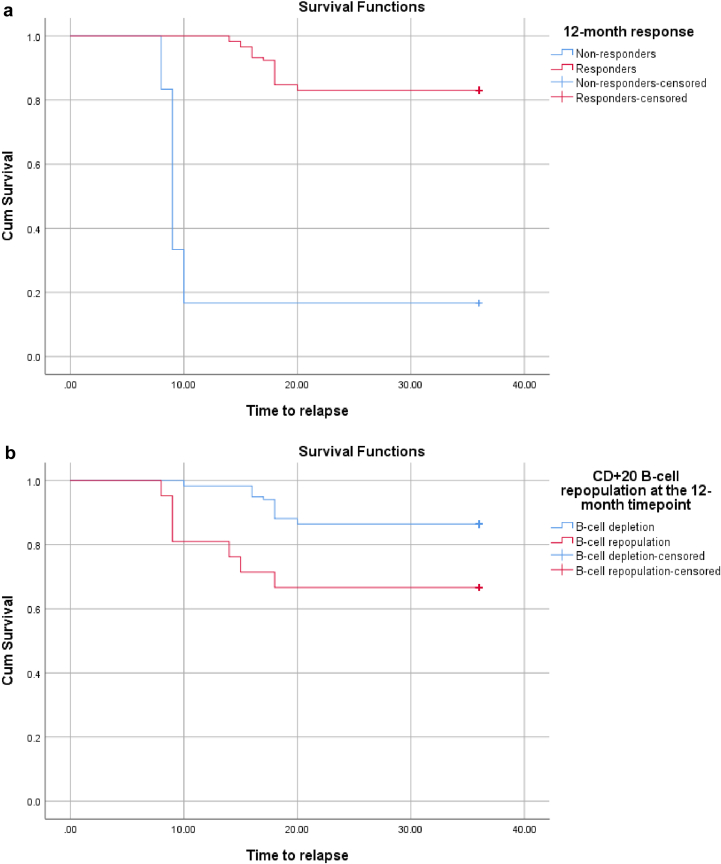
Relapse-free survival in relation to 12-month response and B-cell depletion after RTX. (a) Patients achieving 12-month response showed significantly better relapse-free survival (RFS) (mean: 32.8 months, 83% relapse-free) compared with nonresponders (median: 9 months, 17% relapse-free; log-rank *P* < 0.001). (b) Patients maintaining B-cell depletion at 12 months exhibited significantly superior RFS (86%) versus those with B-cell reconstitution (67%), indicating that sustained B-cell depletion provided superior long-term disease control (log-rank *P* = 0.001). cum, cumulative; RTX, rituximab.

**Figure 4:**
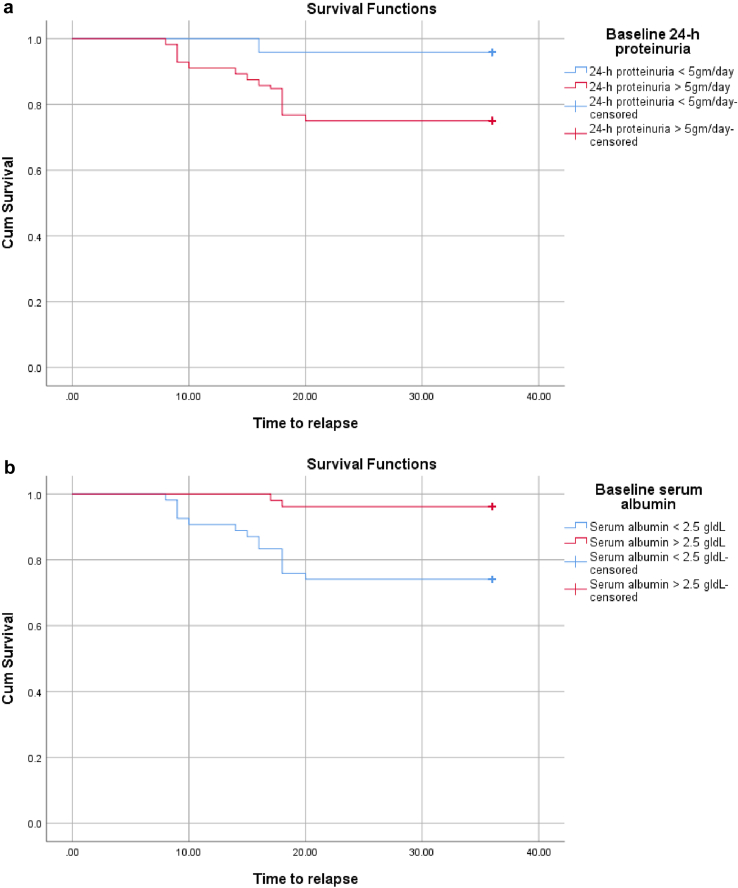
Baseline disease severity as a predictor of relapse-free survival after RTX therapy. (a) Lower baseline proteinuria (< 5 g/d) was significantly associated with a reduced relapse rate following RTX treatment (4% vs. 25%; log-rank *P* = 0.002). (b) Higher baseline serum albumin (≥ 2.5 g/dl) was strongly associated with lower relapse rates (4% vs. 26%; log-rank *P* = 0.001), suggesting that patients with milder disease at presentation had better long-term outcomes. cum, cumulative; RTX, rituximab.

The survival analysis identified key predictors of RFS in RTX-treated patients. A 12-month response was strongly predictive of sustained remission (*P* < 0.001), whereas sustained B-cell depletion at 12 months was associated with superior disease control (*P* = 0.001) (Figure 3). Baseline disease severity markers were most predictive, with patients having proteinuria < 5 g/d (*P* = 0.002) and serum albumin ≥ 2.5 g/dl (*P* = 0.001) showing significantly lower relapse rates (Figure 4). CNI responsiveness played a key role (*P* = 0.002), with outcomes varying by response type: CNI-resistant patients had the poorest prognosis, CNI-dependent patients showed moderate outcomes, and frequent relapsers achieved the most favorable responses. Steroid discontinuation at RTX initiation was associated with better outcomes (*P* = 0.048). Histological variants significantly influenced prognosis (*P* = 0.001), ranging from 100% RFS in tip variant to 33.3% in collapsing variant. RTX dosing regimen, BMI, and steroid responsiveness had no significant impact on outcomes (Supplementary Figures S3–S5).

### RTX Retreatment

A total of 44 patients received a second course of RTX following their initial treatment. Retreatment was equally indicated for persistent disease activity (50%) and relapsing disease (50%), reflecting a cohort with ongoing or recurrent activity. Majority of these patients were nonresponders to the first RTX course (59%), and predominantly SR cases (64%), indicating a particularly refractory population. Both RTX dosing regimens, 1 g 2 weeks apart and 375 mg weekly, were used nearly equally. Histological subtypes were relatively evenly distributed, with NOS and collapsing variants each accounting for 32%, followed by the cellular variant (27%) and the tip variant (9%). Notably, adverse side effects occurred in greater than one-third of patients (36%). Following retreatment, 24 patients remained nonresponders, whereas 18 patients achieved PR, and CR was observed in only 2 patients. The average time to retreatment was approximately 10 months. Overall, the data reflected the complexity and refractory nature of this cohort, with limited success upon retreatment (Supplementary Table S5).

### Long-Term Follow-Up Outcomes Following 12-Month RTX Treatment

Of the 160 patients enrolled, 118 demonstrated treatment response at the 12-month evaluation, whereas 42 patients showed no response, including 32 with ongoing disease activity and 10 who relapsed following initial response. Thirty-two patients received additional RTX treatment, consisting of 22 nonresponders and 10 patients who had relapsed. Postretreatment showed 10 patients achieving PR, whereas 22 patients continued to exhibit persistent disease activity. Among the 118 initial responders, 82 patients sustained remission, with 40 patients achieving CR and 42 patients maintaining PR. However, 20 patients developed disease relapse. Of these relapsers, 12 patients underwent second-course RTX therapy, yielding CR in 2 patients, PR in 8 patients, and treatment failure in 2 patients. Throughout the 24-month observation period, 16 patients were lost to follow-up (Supplementary Figure S6).

## Discussion

Primary FSGS in adults remains challenging to manage because of its diverse features and unclear pathogenesis,^27^ with treatment focused on achieving PR to prevent end-stage kidney disease.^28^ Whereas new antiproteinuric drugs are expanding treatment options, B-cell–targeted immunosuppressive therapies such as RTX represent a favorable treatment approach with a good safety profile,^29^ though the role of RTX in adult FSGS was uncertain despite its proven efficacy in pediatric cases.^30^ This study supports the use of RTX in adult primary FSGS, especially in S-D patients, demonstrating response rates consistent with the existing literature and significant improvements in proteinuria and serum albumin within 12 months. With all responses occurring within this time frame, the findings suggest that 12 months represents an appropriate end point for future FSGS trials. We found that patients with less severe nephrotic syndrome responded better to RTX, specifically those with lower baseline proteinuria and serum creatinine, and higher serum albumin levels. Notably, in patients with low body surface area, proteinuria < 3.5 g/d may still represent nephrotic-range values; however, such cases were not included in this study. The mechanisms behind this association need further investigation, possibly involving milder glomerular injury or better RTX pharmacokinetics in patients with lower proteinuria.^31^^,^^32^ Although this raises questions about RTX dosage effects seen in other glomerulonephritides,^33^^,^^34^ no dose-response association was observed, consistent with Tedesco *et al.*^18^

Our findings are consistent with the literature demonstrating variable RTX effectiveness in FSGS treatment. Several studies have reported favorable outcomes, including Tedesco *et al.*,^18^ who reported response rates of 39%, 52%, and 42% at 3, 6, and 12 months, respectively, with improved results in patients with proteinuria < 5 g/24 h and S-D disease; Roccatello *et al.*,^35^ who documented PR in 5 of 7 patients at 12 months; and Osterholt *et al.*,^23^ who reported 72% CR and 26% PR at 6 months. Conversely, certain studies have reported disappointing RTX outcomes. In the study by Hladunewich *et al.*,^3^ only 2 of 9 patients achieved a short-lived response at 6 months, and all remained nephrotic at 12 months. These contrasting results highlight the heterogeneous nature of RTX therapeutic response in FSGS management. Twelve-month RTX responders had significantly better outcomes with a mean RFS of 32.8 months and a 17% relapse rate versus nonresponders (median: 9 months, 83% relapse rate; *P* < 0.001), establishing 12-month response as a strong predictor of long-term disease control. Consistent with our results, Gauckler *et al.*^26^ conducted a large multicenter retrospective study of adult patients with podocytopathies, and reported that RTX responders achieved superior RFS (hazard ratio: 2.05) and were more likely to remain off additional immunosuppression (61% vs. 36%; OR = 2.69).

A 2020 meta-analysis of 16 observational studies involving 221 adults (51 with FSGS and 170 with minimal change disease [MCD]) found that RTX induced remission in 53.6% of patients with FSGS (42.9% CR), but with a high relapse rate (47.3%) and marked heterogeneity. All studies were uncontrolled, and concomitant use of other immunosuppressants (e.g., steroids, CNIs) varied.^36^ In Tedesco *et al.*,^18^ many adult patients with primary FSGS received RTX in combination with steroids, CNIs, or MMF, either continued from previous treatment or tapered after RTX initiation. By 12 months, the proportion who were off glucocorticoids increased from 42% to 54%, indicating some steroid-sparing effect, although nearly half still required steroids. Exact CNI and MMF use over time was not reported. Remission rates (39% at 3 months, 52% at 6 months, and 42% at 12 months) reflected RTX given alongside ongoing background immunosuppression rather than as monotherapy.^18^ In Gauckler *et al.*,^26^ RTX was most often introduced as an adjunct to existing immunosuppression in adults with primary podocytopathies, including FSGS and MCD; frequently in patients already receiving glucocorticoids, CNIs, or MMF for relapsing or steroid- or CNI-dependent disease. RTX was used to induce remission and facilitate tapering or discontinuation of background agents; and during follow-up, many achieved sustained remission on RTX maintenance alone. However, as with Tedesco *et al.*,^18^ the design did not isolate RTX monotherapy effects, and the observed benefits largely reflect its role in a combined immunosuppression-sparing strategy.^26^

Our analysis found no significant impact of BMI or cumulative RTX dose on RFS or treatment response at 3-, 6-, and 12-month intervals. This aligns with Gauckler *et al.*^26^ and a large retrospective study (*n* = 183) showing no relationship between BMI or RTX dose and remission rates,^23^ suggesting that fixed and weight-adjusted dosing may be equally effective.^26^ However, Kamei *et al.*^37^ found higher remission rates with increased cumulative RTX doses in pediatric or young adult populations, whereas the NEMO study subset analysis suggested that obesity might reduce B-cell depletion efficiency, indicating potential population-specific differences in how BMI and dosing affect outcomes.^38^

S-D patients had higher RTX response rates and comprised more responders, whereas SR patients were predominantly nonresponders, though steroid response status did not significantly affect RFS. S-D FSGS is more immunologically active and responsive to B-cell therapies such as RTX, supported by Ravani *et al.*ʼs^30^ findings of high response rates in S-D children,^39^ whereas SR patients often have severe podocyte injury or structural variants (e.g., collapsing FSGS), causing lower response rates. However, Gauckler *et al.*^26^ found steroid response phenotype did not independently predict long-term relapse-free survival, suggesting that whereas steroid dependence may predict initial response, factors such as B-cell repopulation kinetics, histological subtype, and maintenance therapy are more important for sustained remission. This highlights that initial responsiveness and long-term control are not always aligned in FSGS.^26^ Steroid discontinuation at RTX initiation was significantly associated with better RFS and improved response, with 85% of responders having discontinued steroids versus only 15% who continued at 12 months (*P* = 0.002). Ruggenenti *et al.*^15^ similarly found that steroid discontinuation before RTX linked to improved sustained remission, likely because patients who could safely stop steroids, had more responsive disease or less chronic injury. Retrospective analyses in adult S-D or frequently relapsing FSGS and MCD showed that steroid-free patients at RTX induction had significantly longer RFS and higher CR rates compared with those on chronic steroids, suggesting that steroids may impair RTX’s immunomodulatory effects.^40^

CNI responsiveness strongly predicted RTX outcomes, with CNI-dependent patients showing superior response rates (OR = 5.8, *P* < 0.001) and better RFS (∼ 80%) compared with CNI-resistant patients, who had poor response odds (OR = 0.15, *P* = 0.003) and the worst RFS (50%), whereas frequent CNI relapsers had the best outcomes (∼ 90% RFS). Lin *et al.*’s^42^ study of 42 adults with FSGS or MCD confirmed that CNI-dependent patients and frequent relapsers had more favorable long-term outcomes with higher sustained remission rates, whereas CNI-resistant patients had significantly worse prognoses.^41^ However, Hansrivijit *et al.*’s^36^ meta-analysis contradictorily showed that RTX achieved meaningful remission regardless of previous CNI response. These findings suggest that CNI responsiveness reflects a more immunologically modifiable disease phenotype where B-cell mechanisms predominate, making patients more susceptible to RTX, whereas CNI-resistant patients likely represent structurally or genetically driven FSGS that is less immune-mediated and therefore less RTX-responsive.^41^

We revealed histological variant-specific RTX responses, including tip variant (100% RFS), NOS and cellular variants (81% RFS), and collapsing variant (33% RFS). Collapsing and cellular variants were more common in nonresponders, whereas NOS predominated in responders. The results align with the literature showing that the tip variant has the best prognosis (87% remission),^42^^,^^43^ the collapsing variant is most aggressive (14% RTX response, 33% 3-year survival),^44^ the cellular variant has intermediate outcomes (44%–45% remission, 28% end-stage renal disease progression),^22^ and NOS shows superior RTX response (61.9% CR and 65% RFS).^18^^,^^40^ However, some studies report conflicting results, with up to 60% remission in collapsing FSGS.^45^ This evidence supports the observed NOS predominance among responders and the association of collapsing or cellular variants with therapeutic resistance. Variations between studies reflect differences in cohort characteristics, disease severity, genetics, and treatment protocols.

RTX nonresponders had nearly 8 times higher adverse event rates than responders, with these events strongly associated with poor outcomes and shortened time-to-relapse. Adverse effects may both reflect immune dysregulation and directly impair efficacy by necessitating dose delays, reductions, or discontinuation, leading to incomplete B-cell depletion. Studies by Kamei *et al.*^46^ and Takei *et al.*^47^ found that adverse effects were more common in nonresponders and independently associated with increased relapse rates, indicating that these events serve as both indicators and contributors to poor RTX response. However, contrasting results from Hansrivijit *et al.*’s^36^ meta-analysis of 382 adult patients with MCD or FSGS showed high RTX efficacy (84.2% CR) with minimal adverse events that had no significant correlation with treatment outcomes.

Patients with B-cell recovery at 12 months had markedly inferior outcomes with response rates of 13%, 10%, and 5% at 3, 6, and 12 months, versus 87%, 90%, and 95% in sustained B-cell depletion patients (*P* < 0.001). Furthermore, B-cell repopulation at 6 months predicted poor prognosis (OR = 0.07, *P* = 0.018), and sustained B-cell depletion at 12 months achieved better RFS (86% vs. 67%, *P* = 0.001). Sellier-Leclerc *et al.*^48^ confirmed that prolonged B-cell suppression reduced relapse risk, with CD19+ reappearance marking disease recurrence. However, Kamei *et al.*^49^ found no consistent correlation between peripheral B-cell repopulation and clinical relapse, noting that some patients remained in remission despite early B-cell recovery, suggesting that T-cell dysregulation or podocyte autoimmunity may influence disease activity. Fujinaga *et al.*^50^ observed relapses despite continued B-cell depletion, indicating that peripheral B-cell counts may not reflect tissue-level immune activity.

Pediatric studies offer important guidance for adult FSGS management. The RCRNS01 Japanese randomized controlled trial showed that 4-dose RTX tripled relapse-free intervals (∼11 vs. 3 months) and halved 1-year relapse rates in complicated frequently relapsing or S-D nephrotic syndrome,^51^ whereas an Italian randomized controlled trial found that S-D children had a median time-to-relapse of 18 versus 6 months on steroids, with B-cell reemergence predicting relapse.^52^ A 2023 meta-analysis of 476 pediatric cases confirmed that RTX improves 6 to 12 month RFS and enables steroid sparing, with low proteinuria, preserved albumin, sustained B-cell depletion, and maintenance dosing linked to durable remission in adults.^53^ Sellier-Leclerc *et al.*^48^ reported 68% 12-month sustained remission in steroid- or CNI-dependent children, supporting adult stratification by immunosuppressive dependence, disease duration, relapse frequency, and withdrawal goals, with dependent patients responding better than resistant cases. Girişgen *et al.*^54^ found that 4 weekly RTX doses induced remission in 100% of S-D versus 27% of SR children; CD19+ depletion correlated with remission, and relapse coincided with repopulation, suggesting that early multidose RTX in adults may enhance efficacy but requires B-cell monitoring and individualized dosing. Patients with pediatric-onset FSGS showed significantly superior early RTX response rates compared with adult-onset FSGS at 3 and 6 months, with studies reporting 80% to 100% remission rates in children versus 43% CR and 11% PR in adults,^30^^,^^51^^,^^55^ likely reflecting more immunologically driven and less fibrotic disease in younger patients. However, this advantage diminished by 12 months, when outcomes became comparable across age groups after accounting for disease chronicity, histologic variants, and maintenance immunosuppression.

This largest-to-date study of 160 RTX-treated adult patients with primary FSGS outperforms the previous Italian cohort (31 patients) in scope and depth.^18^ Both confirmed RTX’s efficacy and safety, particularly in S-D patients with lower proteinuria, showing proteinuria and albumin improvements within 6 months. However, retreatment was less beneficial in SR or collapsing variants. The current cohort was younger, more severely ill, with higher proteinuria (8.3 vs. 5.2 g/d), lower albumin (2.17 vs. 2.78 g/dl), and higher steroid resistance (56% vs. 35%), whereas the Italian study included more tip variants and longer disease duration. Superior 12-month response rates (74% vs. 42%), mainly partial remissions, were achieved, with additional independent predictors identified, including steroid dependence, low proteinuria, NOS histology, and sustained B-cell depletion, unlike the Italian study, which identified only steroid dependence and proteinuria significant. This study uniquely analyzed RFS, identifying predictors such as higher albumin, CNI responsiveness, NOS subtype, steroid withdrawal, and absence of B-cell repopulation. Long-term follow-up (160 vs. 11 patients) showed that 69.5% of 12-month responders maintained remission at the 24-month follow-up. Adverse event rates were similar (10% vs. 13%), mostly among nonresponders.

This study has various strengths. It represents the largest cohort available to date and enabled comparisons of response rates across S-D and SR patients within the same cohort. In addition, it allowed for an exploratory assessment of factors related to treatment response. Our study has enhanced the understanding of clinical factors associated with RTX response and RFS by including a larger and more diverse patient population. Previous epidemiological research on RTX efficacy in FSGS treatment has largely been limited to case reports and small case series. In addition, this is the first study to evaluate the effectiveness of RTX in patients with FSGS after excluding those with known genetic variants. Identifying patients with genetic forms of FSGS is crucial, because it guides the selection of appropriate treatment strategies. However, our study has several limitations. Its retrospective design limits data completeness and quality, particularly for patient-reported variables such as low birthweight, which may be subject to recall bias. RTX dosing varied among patients because treating physicians individualized doses according to patient-specific factors and local practice patterns, without adherence to a standardized protocol. Important biological predictors, including anti-RTX antibodies, the Cameron index, and antinephrin antibodies, were not assessed, because these tests were not part of routine practice during the study period. Serum IgG levels were not measured, preventing evaluation of baseline immune status or RTX-induced hypogammaglobulinemia as potential contributors to adverse events or outcomes. In addition, small sample sizes in subgroup analyses reduced statistical power and increased the risk of type II errors, potentially limiting the generalizability of the findings.

In conclusion, RTX is a safe and effective therapy for adult primary FSGS, especially in S-D and CNI-responsive patients, with most achieving remission within 12 months and maintaining sustained RFS. This study is the first to comprehensively identify independent predictors of RTX response and RFS, including lower baseline proteinuria, higher serum albumin, NOS histology, absence of B-cell repopulation, steroid withdrawal, and CNI responsiveness. Although RTX showed limited efficacy in SR and collapsing variants, BMI and RTX dose did not influence outcomes, supporting fixed dosing. Adverse events were recorded in 16 patients, occurred more frequently among nonresponders and correlated with poorer outcomes. RTX enabled steroid sparing, benefiting treatment responders. Twelve-month responders had significantly longer RFS (83% relapse-free) compared with nonresponders (17% relapse-free). Conversely, early B-cell repopulation, ongoing steroid use, and collapsing histology were associated with inferior RFS.

## Disclosure

All the authors declared no competing interests.

## Supplementary Material

Supplementary File (PDF) **Supplementary Methods.** Comprehensive laboratory and imaging workup. **Figure S1.** Different variants of primary FSGS. **Figure S2.** Changes in proteinuria, serum albumin, and serum creatinine in all studied patients at 12 months. **Figure S3.** Influence of steroid and calcineurin inhibitor responsiveness on relapse-free survival. **Figure S4.** Prognostic significance of rituximab adverse events and FSGS histological variants in relapse-free survival. **Figure S5.** Impact of rituximab regimen, treatment indication and body mass index on relapse-free survival. **Figure S6.** Long-term outcomes and retreatment response following initial 12-month RTX therapy. **Table S1.** Association between BMI, concomitant steroid use at RTX initiation, cumulative RTX dose, and treatment response at 3, 6, and 12 months. **Table S2.** RTX-associated adverse side effects in the study population. **Table S3.** CD20+ B-cell repopulation rates and their association with RTX response. **Table S4.** Univariate logistic regression determining predictors of 12-month RTX response. **Table S5.** Clinical characteristics of patients undergoing RTX retreatment after the initial therapy.
