# Supplementary material for: Efficacy of Rituximab as Adjunctive Therapy to Immunosuppressive Agents in Adult Primary Focal Segmental Glomerulosclerosis
Source: Kidney Int Rep. 2025 Sep 3;10(11):3918–36. doi: 10.1016/j.ekir.2025.08.046 (PMC12640038; doi:10.1016/j.ekir.2025.08.046)
Supplement: Supplementary File (PDF) — Supplementary Methods. Comprehensive laboratory and imaging workup. Figure S1. Different variants of primary FSGS. Figure S2. Changes in proteinuria, serum albumin, and serum creatinine in all studied patients at 12 months. Figure S3. Influence of steroid and calcineurin inhibitor responsiveness on relapse-free survival. Figure S4. Prognostic significance of rituximab adverse events and FSGS histological variants in relapse-free survival. Figure S5. Impact of rituximab regimen, treatment indication and body mass index on relapse-free survival. Figure S6. Long-term outcomes and retreatment response following initial 12-month RTX therapy. Table S1. Association between BMI, concomitant steroid use at RTX initiation, cumulative RTX dose, and treatment response at 3, 6, and 12 months. Table S2. RTX-associated adverse side effects in the study population. Table S3. CD20+ B-cell repopulation rates and their association with RTX response. Table S4. Univariate logistic regression determining predictors of 12-month RTX response. Table S5. Clinical characteristics of patients undergoing RTX retreatment after the initial therapy. [file mmc1.pdf]

Supplemental Methods

Comprehensive laboratory and imaging workup

After a 9-hour fast, 12 mL of venous blood had been drawn under aseptic conditions as part of routine clinical evaluation and processed into multiple portions: 2 mL in EDTA tubes for CBC (automated cell counter; CelltacES, Nihon Kohden, Japan), 6 mL in plain tubes for serum-based testing (stored at −70 °C), and 4 mL for immunological and infectious screening. Results for fasting glucose and HbA1c (Audicom AC9900 Automatic Electrolyte Analyzer, China); renal and liver function tests, albumin, cholesterol, and triglycerides (SELECTRA PRO XL auto-analyzer, ELITech Group, Netherlands); and viral markers (HCV, HBsAg, HIV) assessed using cobas e 411 (ELITech Group, Germany) were retrieved. C-reactive protein results obtained by latex agglutination (Biomedical Systems) and serum protein electrophoresis performed on agarose gel were recorded. Antinuclear antibodies (ANA), C3, and C4 levels measured with cobas c11 (Hitachi High Technologies Corporation, Japan) and quantitative PCR results for Parvovirus B19, EBV, and CMV DNA were also reviewed. Urine dipstick testing and 24-hour protein quantification by turbidimetry, as well as Bence Jones protein detection via immunofixation electrophoresis, were documented. For patients with tuberculosis-suggestive symptoms, results from sputum PCR with the Xpert MTB/RIF assay (Cepheid, Sunnyvale, CA, USA) were reviewed. Where available, results of genetic testing by targeted next-generation sequencing, performed in SR patients and those with familial or syndromic features, were recorded to exclude genetic FSGS.

Comprehensive two- and three-dimensional transthoracic echocardiography results were reviewed from assessments performed prior to RTX administration to evaluate heart failure (HF), exclude congenital cyanotic heart diseases that may cause secondary FSGS, and identify potential RTX-related cardiac complications. The echocardiographic assessment included left ventricular ejection fraction (LVEF) measurement using the Simpson biplane and M-mode techniques, analysis of segmental wall motion abnormalities, diastolic function evaluation through tissue Doppler and mitral inflow Doppler, assessment of ventricular dimensions and volumes, and detection of ischemic mitral regurgitation. HF was defined as a clinical syndrome characterized by impaired ventricular filling or ejection, and was classified into two main categories: HF with reduced ejection fraction (HFrEF, LVEF <40%) and HF with preserved ejection fraction (HFpEF, LVEF ≥50%). HFrEF was diagnosed when HF symptoms coexisted with an LVEF <40%. HFpEF diagnosis required HF symptoms along with an LVEF ≥50% and additional evidence of diastolic dysfunction or structural heart disease, including LV hypertrophy (mass index ≥115 g/m^2 for men, ≥95 g/m^2 for women), left atrial dilation (volume index ≥34 mL/m^2), or an elevated E/e′ ratio (≥13).

Kidney biopsy findings were obtained from pathology reports of percutaneous biopsies performed using automated biopsy guns under ultrasound guidance. Tissue samples were processed for LM and EM using 10% neutral buffered formalin and EM using 2.5% glutaraldehyde. For LM, 3-μm paraffin-embedded sections were stained with hematoxylin and eosin (H&E), periodic acid–Schiff (PAS), Masson trichrome, and Jones methenamine silver, and examined using an Olympus BX50 microscope at 10×, 200×, and 400× magnifications. FSGS variants (not otherwise specified [NOS], tip, collapsing, and cellular) were diagnosed, according to histopathology reports, and glomerular changes, including mesangial sclerosis, tuft collapse, epithelial hyperplasia, and inflammatory cell infiltration, were documented. EM findings for mesangial sclerosis, podocyte effacement, and GBM alterations were also recorded.

Supplemental Tables

Supplemental Table S1. Association between BMI, concomitant steroid use at RTX initiation, cumulative RTX dose, and treatment response at 3, 6, and 12 months.

**Subgroups**	**Response**		
	**3 months (*n*= 92)**	**6 months (*n*= 114)**	**12 months (*n*= 118)**
**BMI ≥ 25 kg/m^2**	42 (46%)	50 (44%)	56 (47%)
**BMI < 25 kg/m^2**	50 (54%)	64 (56%)	62 (53%)
P value	0.86	0.34	0.61
**Concomitant steroid therapy at RTX therapy**	16 (17%)	22 (19%)	18 (15%)
**Discontinued steroid therapy at RTX therapy**	76 (83%)	92 (81%)	100 (85%)
P value	0.17	0.34	**0.002**
**Cumulative RTX dose ≥ 2g**	44 (48%)	52 (46%)	54 (46%)
**Cumulative RTX dose < 2g**	48 (52%)	62 (54%)	64 (54%)
P value	0.92	0.45	0.46

BMI, Body mass index; RTX, Rituximab. The categorical variables are expressed as number (percentage).

Supplemental Table S2. RTX-associated adverse side effects in the study population

Adverse event	Number of events (%)	Number of patients affected	Hospitalization required	Severity	Management &Outcome
Infections (Total)	16 (54%)	8	7	Mild to severe	Antibiotics (IV/Oral), supportive care. All recovered
• Sepsis	4	2	4	Severe	IV antibiotics, inpatient care. Recovered
• Pneumonia	5	3	3	Moderate to severe	Oral/IV antibiotics. Recovered
• UTI	7	3	0	Mild	Oral antibiotics, outpatient. Recovered
Infusion reactions	10 (33%)	6	2	Mild (8), severe (2)	Slowing infusion, antipyretics, antihistamines, corticosteroids. Resolved, no ICU required
Myocarditis	4 (13%)	2	4	Moderate to severe	Full recovery, no HF
Total	30 (100%)	16	13	-	No deaths; 10 relapsed, 6 had PR

HF, Heart failure; ICU, Intensive care unit; PR, Partial remission; UTI, Urinary tract infection.

Supplemental Table S3. CD20+ B-cell repopulation rates and their association with RTX response

Month of CD20 count evaluation	CD20+ B-cells repopulation	Response		
		**3 months**	**6 months**	**12 months**
3 months	Repopulated	0 (0%)	0 (0%)	0 (0%)
	Not repopulated	92 (100%)	114 (100%)	118 (100%)
	P Value	**< 0.001**	**< 0.001**	**< 0.001**
6 months	Repopulated	6 (6%)	6 (5%)	6 (5%)
	Not repopulated	86 (94%)	108 (95%)	112 (95%)
	P value	**< 0.001**	**< 0.001**	**< 0.001**
12 months	Repopulated	12 (13%)	12 (10%)	6 (5%)
	Not repopulated	80 (87%)	102 (90%)	112 (95%)
	P value	**< 0.001**	**< 0.001**	**< 0.001**

The categorical variables are expressed as number (percentage).

Supplemental Table S4. Univariate logistic regression determining predictors of 12-month RTX response

Variables	Univariate logistic regression			
	P	**Odds ratio (OR)**	**95% confidence interval (CI)**	
			**Lower bound**	**Uppper bound**
HTN	< **0.001**	0.16	0.07	0.36
Baseline serum creatinine	< **0.001**	0.36	0.23	0.55
Baseline eGFR	< **0.001**	1.02	1.01	1.03
Baseline serum albumin	< **0.001**	4.95	2.27	10.82
Baseline proteinuria	< **0.001**	0.78	0.69	0.87
Steroid status (steroid dependency)	**0.003**	3.31	1.49	7.34
Steroid discontinuation at RTX initiation	**0.003**	3.42	1.54	7.61
Steroid dose at RTX intiation	**0.007**	0.96	0.93	0.99
Prior immunosuppressive therapy (CNI)	< **0.001**	4.80	2.24	10.29
CNI status				
• CNI dependence	< **0.001**	5.8	2.27	14.79
• CNI resistance	**0.003**	0.15	0.04	0.53
MMF resistance	< **0.001**	0.21	0.09	0.45
Histological variants				
• NOS	< **0.001**	9.39	4.13	21.36
• Cellular	**0.005**	0.34	0.16	0.73
• Collapsing	0.99	0.00	0.00	0.00
CD20+ B-cell repopulation at 6 months	< **0.001**	0.04	0.01	0.11
RTX indication (relapses)	**0.013**	0.39	0.19	0.82
RTX side effects	< **0.001**	0.09	0.03	0.29

CNI, Calcineurin inhibitor; DBP, Diastolic blood pressure; eGFR, Estimated glomerular filtration rate; MMF, Mycophenolate mofetil; NOS, Not otherwise specified; OR, Odds ratio; RTX, Rituximab; SBP, Systolic blood pressure. Each 0.1 mg/dL increase in serum creainine was significantly associated with reduced odds of response. Per 0.1 g/dL increase in serum albumin, there was a significant increase in the odds of response. For each 1 g/day increase in proteinuria, the odds of response significantly decreased. Per 1 mL/min/1.73 m² increase in eGFR, there was a significant increase in the odds of response. Each 1 mg/day increase in steroid dose at RTX initiation was associated with a small but significant decrease in odds of response.

Supplemental Table S5. Clinical characteristics of patients undergoing RTX retreatment after the initial therapy.

**Variables**	**Patients retreated with RTX** *n*= 44
Response to first RTX administration	
• NR	26 (59%)
• PR	6 (14%)
• CR	12 (27%)
RTX dosing	
• 1 g two weeks apart	24 (55%)
• 375mg every week	20 (45%)
Steroid status	
• SR	28 (64%)
• SD	16 (36%)
Histological variants	
• NOS	14 (32%)
• Cellular	12 (27%)
• Collapsing	14 (32%)
• Tip	4 (9%)
Indications of RTX retreatment	
• Persistent activity	22 (50%)
• Relapsing disease	22 (50%)
RTX adverse side effects	16 (36%)
Response to retreatment	
• NR	24 (55%)
• PR	18 (41%)
• CR	2 (4%)
Time to retreatment (months)	
Mean ± SD	9.86 (4.81)
Range	4 – 20

CR, Complete response; NOS, Not otherwise specified; NR, No response; PR, Partial response; RTX, Rituximab; SD, Standard deviation; SD, Steroid dependent; SR, Steroid resistant. Data are expressed as n (%).

Supplemental Figures

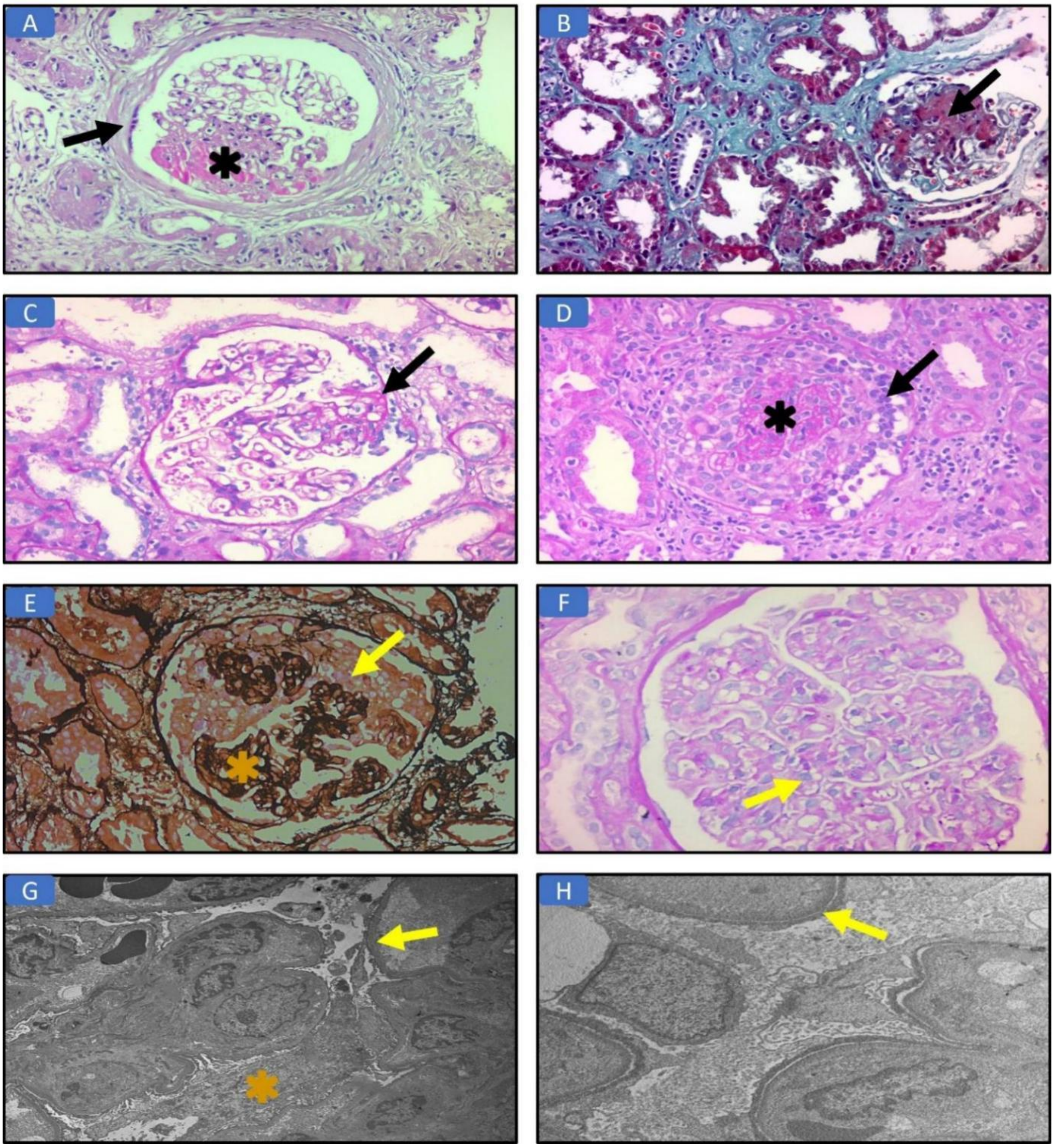

Supplemental Figure S1. Different variants of primary FSGS: (A) FSGS; not otherwise specified (NOS), the glomerulus showed segmental tuft sclerosis and hyalinosis, located randomly within the tufts (asterisk) and periglomerular fibrosis (arrow) (H&E, ×200). (**B) FSGS; NOS,** the glomerulus showed segmental tuft sclerosis and hyalinosis (arrow) and the surrounding interstitium showed patchy fibrosis (masson trichrome, ×100). (**C) FSGS; tip variant,** the glomerulus showed segmental tuft proliferation located at the tip with delicate capsular adhesions (arrow) (PAS, ×200). (**D) FSGS; collapsing variant,** the glomerulus showed segmental tuft collapse (asterisk) with overlying florid epithelial cell hyperplasia (arrow) (PAS, ×200). (**E) FSGS; collapsing variant,** the glomerulus showed segmental tuft collapse (asterisk) with overlying florid epithelial cell hyperplasia (arrow) (JMS, ×200). (**F) FSGS; cellular variant,** the glomerulus showed segmental endocapillary proliferation with infiltrating leucocytes and foam cells (PAS, ×400). (**G and H) Electron microscopy findings of primary FSGS**, the podocytes overlying glomerular basement membranes showed diffuse marked effacement of their foot processes (arrows) with sclerosis in the mesangial area (asterisk) (EM, ×2500).

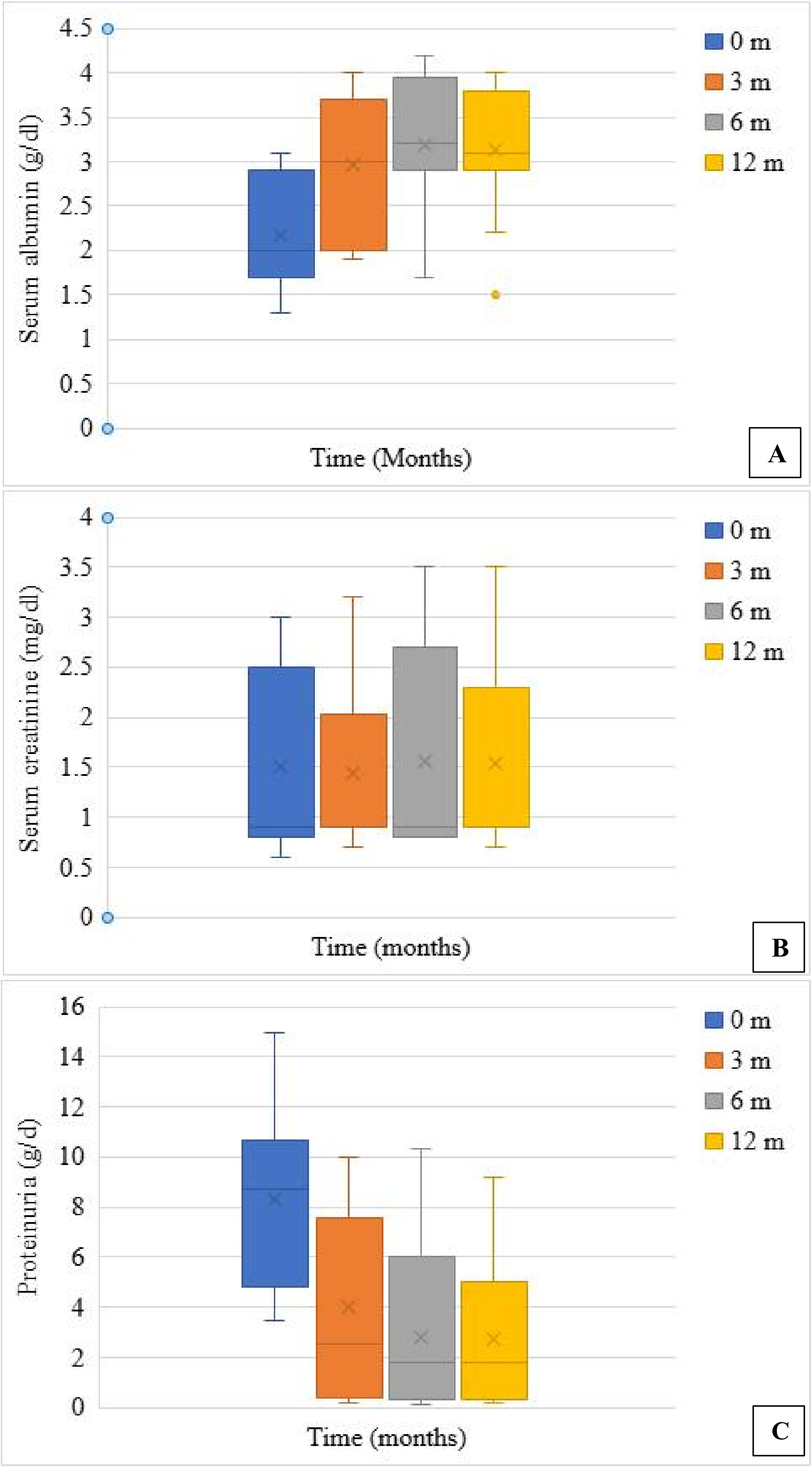

Supplemental Figure S2. Changes in serum albumin, serum creatinine and 24-hour proteinuria in the all studied patients at 12 months. (**A**) Serum albumin levels increased considerably compared to baseline level at 3, 6 and 12 months ($P < 0.001$ for each). (**B**) Serum creatinine levels remained stable during the follow-up period. (**C**) 24-hour proteinuria was significantly decreased at all time points in comparison to baseline level ($P < 0.001$ for each).

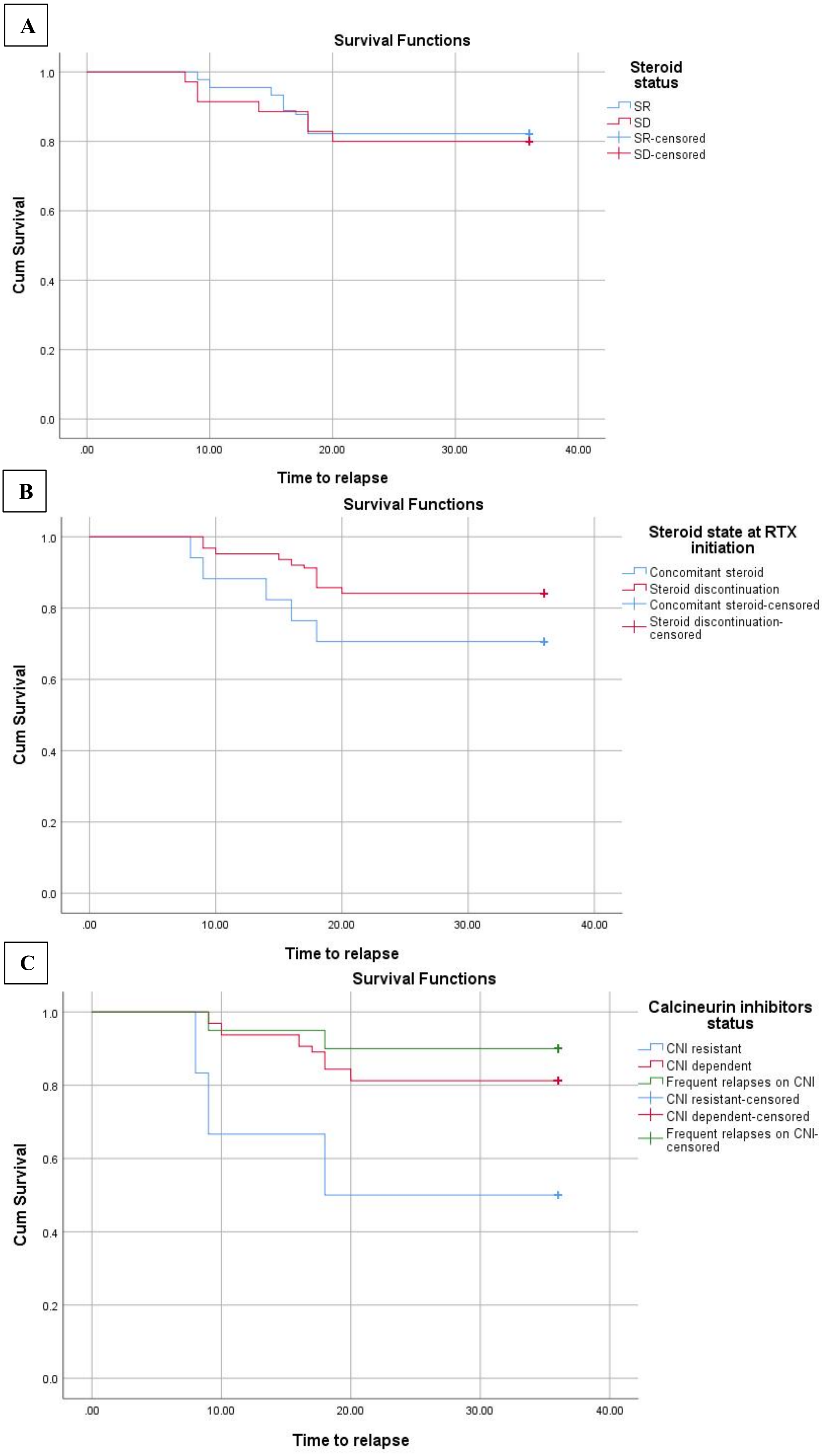
A
Survival Functions
Cum Survival
1.0
0.8
0.6
0.4
0.2
0.0
.00
10.00
20.00
30.00
40.00
Time to relapse
Steroid status
SR
SD
SR-censored
SD-censored
B
Survival Functions
Cum Survival
Time to relapse
Steroid state at RTX initiation
Concomitant steroid
Steroid discontinuation
Concomitant steroid-censored
Steroid discontinuation-censored
C
Survival Functions
Cum Survival
Time to relapse
Calcineurin inhibitors status
CNI resistant
CNI dependent
Frequent relapses on CNI
CNI resistant-censored
CNI dependent-censored
Frequent relapses on CNI-censored

Supplemental Figure S3. Influence of steroid and calcineurin inhibitor (CNI) responsiveness on relapse-free survival (RFS). (**A**) Steroid responsiveness status (steroid resistant vs. steroid dependent) had no significant impact on RFS. (**B**) Patients with steroid discontinuation at RTX initiation had better RFS compared to those on concomitant steroids (Log-rank P = 0.048). (**C**) CNI responsiveness strongly predicted RFS (log-rank P = 0.002), with CNI-resistant patients having the worst outcomes (50% relapse rate), CNI-dependent patients showing intermediate survival (~80%), and frequent relapsers on CNI paradoxically demonstrating the best outcomes (~90% survival).

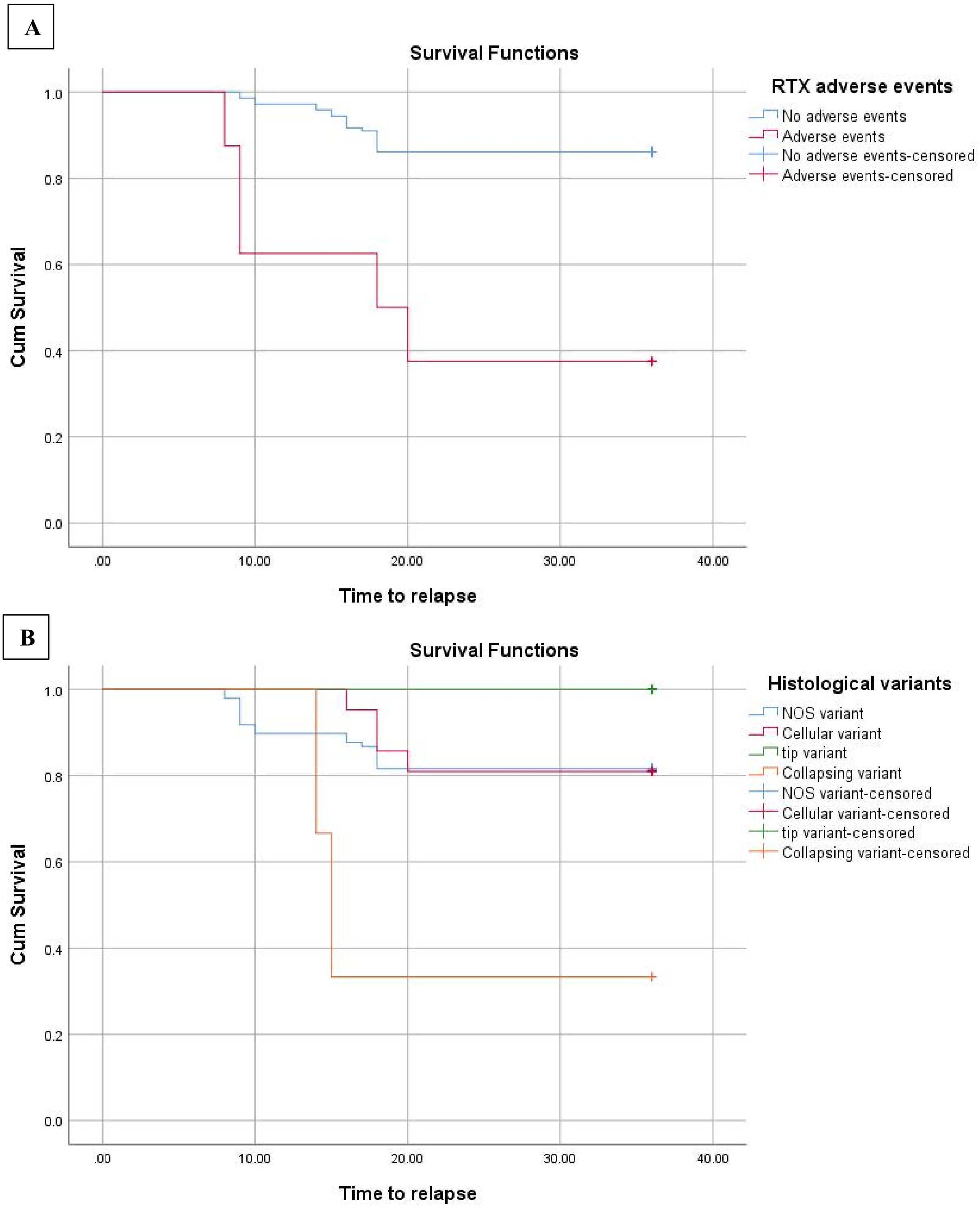

Supplemental Figure S4. Prognostic significance of rituximab adverse events and FSGS histological variants in relapse-free survival (RFS). (**A**) Patients who experienced rituximab adverse events demonstrated notably shorter RFS compared to those without adverse events (Log-rank $P < 0.001$). (**B**) The survival analysis showed significant differences in RFS between histological variants (log-rank $P = 0.001$). The tip variant had the best outcomes (100% RFS), followed by NOS (82%) and cellular (81%) variants, while the collapsing variant had the worst prognosis (33% RFS).

A

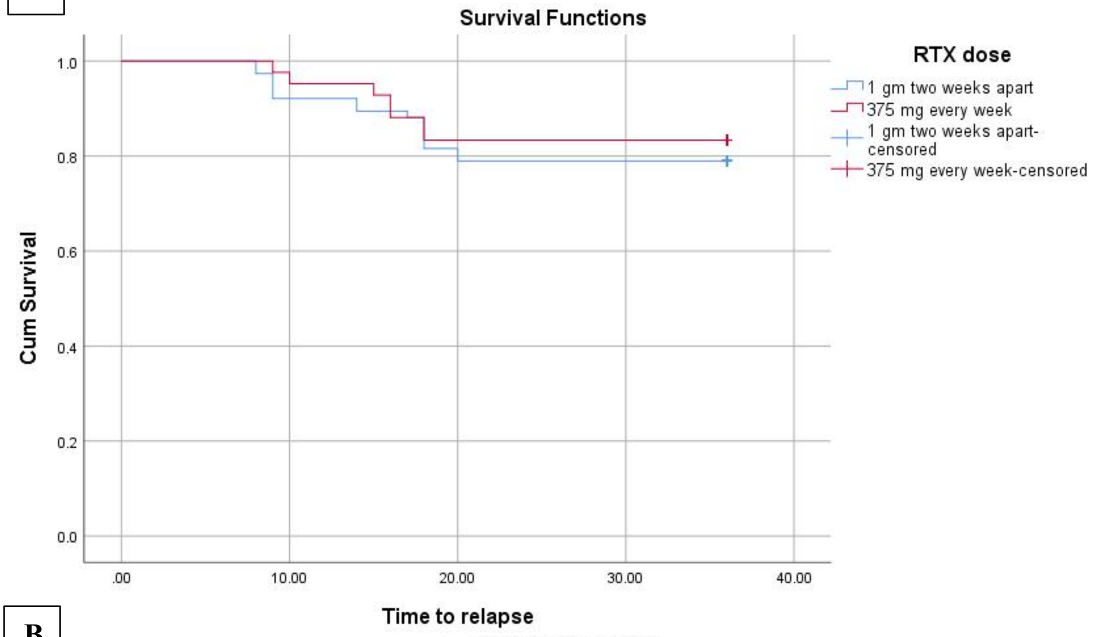

Survival Functions
Cum Survival
1.0
0.8
0.6
0.4
0.2
0.0
.00
10.00
20.00
30.00
40.00
Time to relapse
RTX dose
1 gm two weeks apart
375 mg every week
1 gm two weeks apart-censored
375 mg every week-censored

B

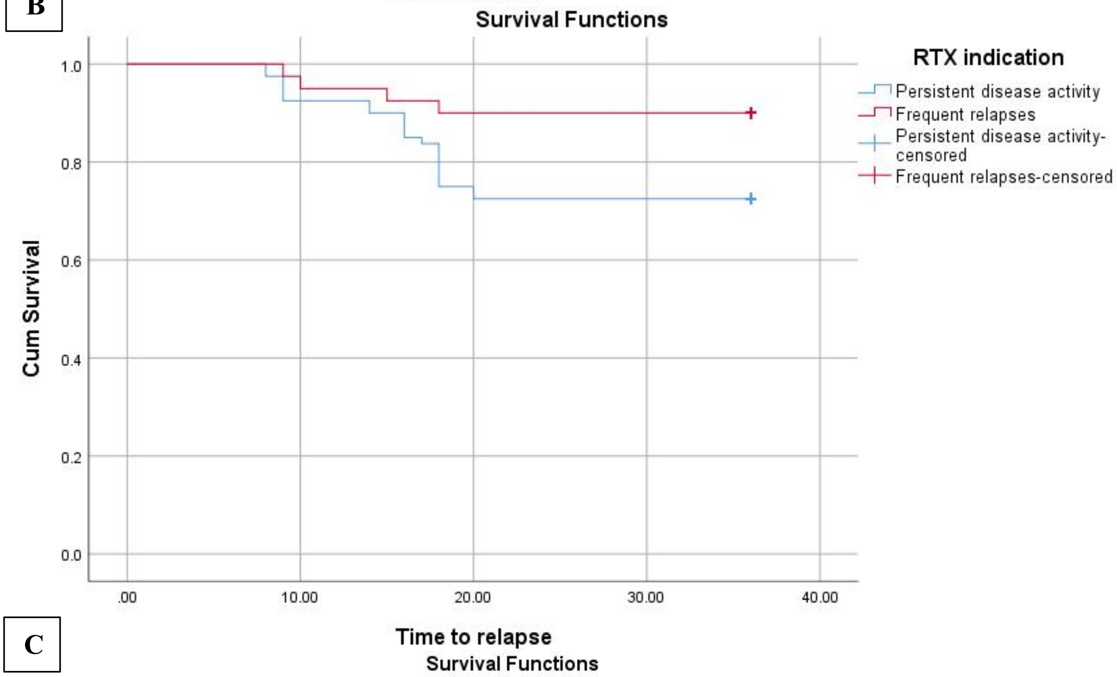

Survival Functions
Cum Survival
1.0
0.8
0.6
0.4
0.2
0.0
.00
10.00
20.00
30.00
40.00
Time to relapse
RTX indication
Persistent disease activity
Frequent relapses
Persistent disease activity-censored
Frequent relapses-censored

C

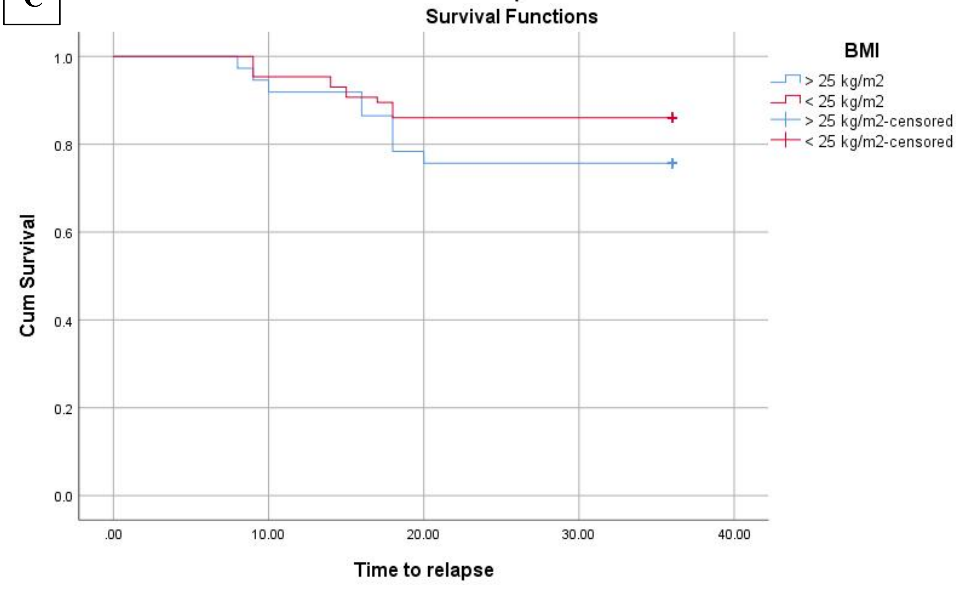

Survival Functions
Cum Survival
1.0
0.8
0.6
0.4
0.2
0.0
.00
10.00
20.00
30.00
40.00
Time to relapse
BMI
> 25 kg/m2
< 25 kg/m2
> 25 kg/m2-censored
< 25 kg/m2-censored

Supplemental Figure S5. Impact of rituximab (RTX) regimen, treatment indication and body mass index (BMI) on relapse-free survival (RFS). (**A**) RTX dosing regimen (375 mg/m² weekly versus 1 g two weeks apart) did not significantly impact RFS outcomes (Log-rank *P* = 0.48). (**B**) Frequent relapses as an indication for RTX therapy was associated with superior RFS compared to persistent disease activity (Log-rank *P* = 0.006). (**C**) BMI showed no impact on RFS based on the overlapping survival curves across all BMI groups (Log-rank *P* = 0.11).

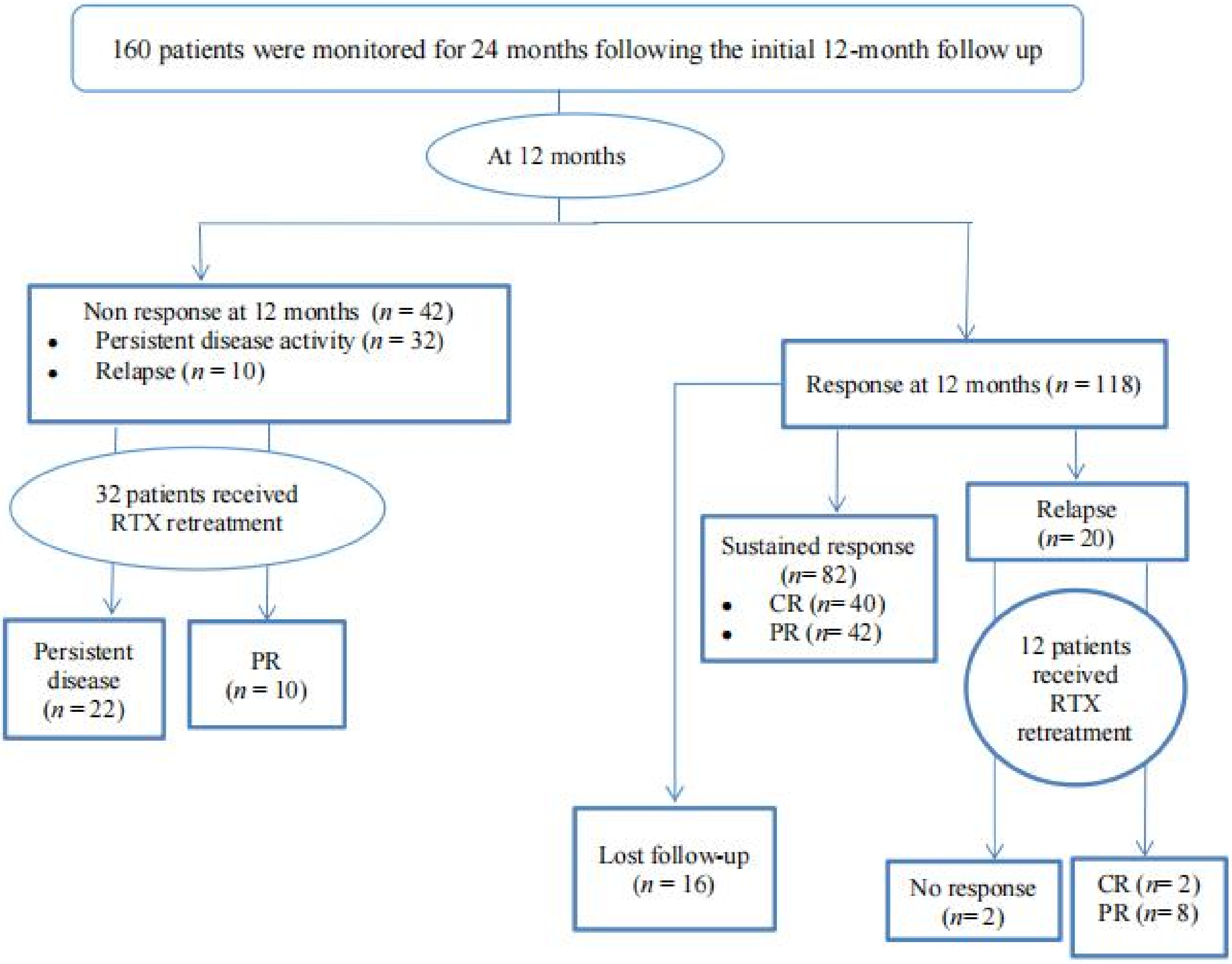

Supplemental Figure S6. Long-term outcomes and retreatment response following initial 12-month RTX therapy.
